# Cohesive Properties of the *Caulobacter crescentus* Holdfast Adhesin Are Regulated by a Novel c-di-GMP Effector Protein

**DOI:** 10.1128/mBio.00294-17

**Published:** 2017-03-21

**Authors:** Kathrin S. Sprecher, Isabelle Hug, Jutta Nesper, Eva Potthoff, Mohamed-Ali Mahi, Matteo Sangermani, Volkhard Kaever, Torsten Schwede, Julia Vorholt, Urs Jenal

**Affiliations:** a Focal Area of Infection Biology, Biozentrum, University of Basel, Basel, Switzerland; b Institute of Microbiology, ETH Zurich, Zurich, Switzerland; c Focal Area of Computational & Systems Biology, Biozentrum, University of Basel, Basel, Switzerland; d Research Core Unit Metabolomics and Institute of Pharmacology, Hannover Medical School, Hannover, Germany; Max Planck Institute for Terrestrial Microbiology

## Abstract

When encountering surfaces, many bacteria produce adhesins to facilitate their initial attachment and to irreversibly glue themselves to the solid substrate. A central molecule regulating the processes of this motile-sessile transition is the second messenger c-di-GMP, which stimulates the production of a variety of exopolysaccharide adhesins in different bacterial model organisms. In *Caulobacter crescentus*, c-di-GMP regulates the synthesis of the polar holdfast adhesin during the cell cycle, yet the molecular and cellular details of this control are currently unknown. Here we identify HfsK, a member of a versatile *N*-acetyltransferase family, as a novel c-di-GMP effector involved in holdfast biogenesis. Cells lacking HfsK form highly malleable holdfast structures with reduced adhesive strength that cannot support surface colonization. We present indirect evidence that HfsK modifies the polysaccharide component of holdfast to buttress its cohesive properties. HfsK is a soluble protein but associates with the cell membrane during most of the cell cycle. Coincident with peak c-di-GMP levels during the *C. crescentus* cell cycle, HfsK relocalizes to the cytosol in a c-di-GMP-dependent manner. Our results indicate that this c-di-GMP-mediated dynamic positioning controls HfsK activity, leading to its inactivation at high c-di-GMP levels. A short C-terminal extension is essential for the membrane association, c-di-GMP binding, and activity of HfsK. We propose a model in which c-di-GMP binding leads to the dispersal and inactivation of HfsK as part of holdfast biogenesis progression.

## IMPORTANCE

Exopolysaccharide (EPS) adhesins are important determinants of bacterial surface colonization and biofilm formation. Biofilms are a major cause of chronic infections and are responsible for biofouling on water-exposed surfaces. To tackle these problems, it is essential to dissect the processes leading to surface colonization at the molecular and cellular levels. Here we describe a novel c-di-GMP effector, HfsK, that contributes to the cohesive properties and stability of the holdfast adhesin in *C. crescentus*. We demonstrate for the first time that c-di-GMP, in addition to its role in the regulation of the rate of EPS production, also modulates the physicochemical properties of bacterial adhesins. By demonstrating how c-di-GMP coordinates the activity and subcellular localization of HfsK, we provide a novel understanding of the cellular processes involved in adhesin biogenesis control. Homologs of HfsK are found in representatives of different bacterial phyla, suggesting that they play important roles in various EPS synthesis systems.

## INTRODUCTION

Microorganisms are predominantly surface associated and often grow in complex multicellular structures called biofilm (1, 2). At the same time, they are able to disperse as motile single cells and explore their environment (3, 4). To effectively switch between these fundamentally different lifestyles, many bacteria have evolved regulatory mechanisms that robustly promote cellular processes associated with motility and sessility, respectively. The ubiquitous second messenger c-di-GMP plays a central role in this transition (5). While c-di-GMP interferes with flagellum- and pilus-based motility (6, 7), it stimulates the synthesis of adhesion factors and extracellular matrix components like curli fibers or exopolysaccharides (EPSs) (8–10). A prime example of c-di-GMP-mediated control is the production of cellulose, a secreted glucose polymer mediating surface attachment and biofilm stability in many bacteria (11). Cellulose is synthesized and translocated through the cell envelope by the membrane-integral BcsAB complex (12). The synthase BcsA is held in an autoinhibitory state by a gating loop that blocks the access of glucose monomers to the catalytic site and that is released upon c-di-GMP binding (13, 14). Similarly, the synthesis of poly-β-1,6-*N*-acetylglucosamine in *Escherichia coli* requires the simultaneous binding of c-di-GMP to the synthase PgaC and to its cosynthase PgaD to stabilize their interaction and boost their activity (15).

We use *Caulobacter crescentus* as a model to study the regulatory mechanisms of the motile-to-sessile transition of bacteria. This Gram-negative freshwater bacterium has a biphasic cell cycle with an asymmetric division producing motile, replication-inert swarmer (SW) cells and sessile, replication-competent stalked (ST) cells (16). SW cells are equipped with a flagellar motor and adhesive pili and remain motile for an extended period before differentiating into ST cells. During this process, they replace their flagellum and pili with an EPS adhesin, the holdfast, which is located at the tip of a cell extension, the stalk. The holdfast, which consists of EPS (17, 18) and additional, undefined components (18–20), mediates strong and permanent attachment of ST cells to surfaces (21–23). The holdfast EPS is composed of oligomers of *N*-acetylglucosamine and is synthesized and anchored by the holdfast synthesis (Hfs) and holdfast anchoring (Hfa) proteins, most of which are encoded in two separate operons in the *C. crescentus* genome (23–25). On the basis of homology models and deletion studies, several glycosyltransferases were predicted to participate in the assembly of a glycosyl oligomer onto a lipid anchor (23, 26, 27). The sugar moieties were proposed to be chemically modified. For example, HfsH is thought to deacetylate a glycosyl subunit(s) of the growing polymer (19). The lipid-linked oligomers are then flipped through the cytoplasmic membrane into the periplasm, further polymerized, and exported to the cell surface (23). Mutants that lack the anchor protein HfaA, -B, or -D shed their holdfast. How these proteins contribute to EPS anchoring is not understood (25, 28).

*C. crescentus* cell morphogenesis and behavior are regulated by c-di-GMP, the levels of which oscillate through the cell cycle (29, 30). The c-di-GMP concentration is low in SW cells, peaks during the SW-to-ST-cell transition, and later becomes intermediate in dividing cells (29, 31). Changes in the c-di-GMP concentration are mediated by cell type-specific diguanylate cyclases (DGCs) and phosphodiesterases (PDEs). While c-di-GMP levels are kept low in SW cells by the PDE PdeA, the c-di-GMP upshift during cell differentiation is mediated by the specific degradation of PdeA (32) and the consecutive activation of PleD, a DGC that is active only in the sessile cell type (33, 34). The upshift of c-di-GMP during cell differentiation leads to ejection of the flagellum (35), stimulates the assembly of the stalk, and prompts the biogenesis of the holdfast adhesin (29). However, how c-di-GMP stimulates these processes has remained unclear.

Here we identify the acetyltransferase HfsK as a novel c-di-GMP effector protein that is required for the formation of a cohesive and stably anchored holdfast. Cells harboring an *hfsK* deletion shed abnormal holdfasts that formed elastic filamentous structures when subjected to shear stress. We show that HfsK activity depends on its association with the cytoplasmic membrane. HfsK remains membrane associated throughout most of the cell cycle but is released into the cytoplasm in a c-di-GMP-dependent manner during the SW-to-ST transition, coinciding with peak c-di-GMP concentrations and with holdfast assembly. We identify a short 25-amino-acid stretch at the C terminus of HfsK as a critical determinant of c-di-GMP binding, membrane association, and protein function. On the basis of our data, we propose that c-di-GMP controls HfsK by coupling its activity to its membrane compartmentalization.

## RESULTS

### CC3689 is a novel c-di-GMP binding protein.

We have recently described capture compound-coupled mass spectrometry (CCMS) technology, a biochemical method to isolate c-di-GMP binding proteins (36). Using CCMS, we isolated an uncharacterized protein (CC3689) directly from *C. crescentus* cell extracts (Table 1). Structure-based homology searches with HHpred (37) revealed that CC3689 belongs to the Gcn5-related *N*-acetyltransferase (GNAT) family, a ubiquitous group of *N*-acyltransferases that acylate a variety of different substrates, ranging from proteins to polyamines and aminoglycosides (38).

**TABLE 1:** HfsK and CC1244 detection by CCMS screening for c-di-GMP effectors

Protein and expt^a^	No. of spectral counts of identified peptides (CCMS experiment/CCMS competition)^b^	
	Soluble fraction	Membrane fraction
HfsK (CC3689)		
1	9/0	14/5
2	8/0	13/4
3	10/0	14/4
CC1244		
1	4/0	1/0
2	0/0	2/1
3	0/0	8/3

^a^ Results of three independent experiments using 10 μM (soluble fraction) or 8 μM (membrane fraction) c-di-GMP capture compound are shown.

^b^ Competition experiments were performed in the presence of 1 mM c-di-GMP.

**FIG 1:**
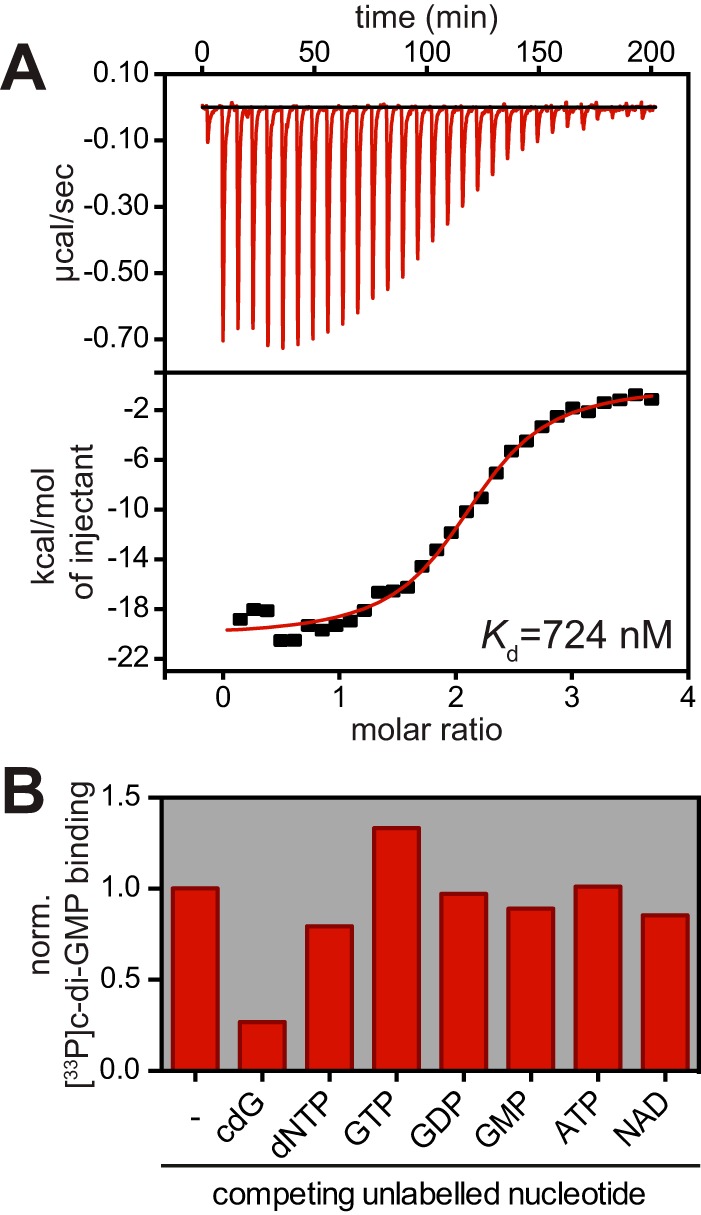
HfsK specifically binds c-di-GMP. (A) ITC measuring the interaction of His-HfsK with c-di-GMP. Heat release upon ligand injection (top), integrated heat plotted against the molar ratio of ligand and protein (bottom), and the resulting disassociation constant (*K*_*d*_) are shown. (B) Quantified autoradiographs of 1 μM purified His-HfsK that has been UV cross-linked to 10 μM [^33^P]c-di-GMP in the presence or absence of a 10-fold excess of nonlabeled nucleotides to test competition. norm., normalized.

To confirm the binding of c-di-GMP to CC3689, we affinity purified a His-CC3689 fusion protein and used it for isothermal titration calorimetry (ITC). His-CC3689 bound c-di-GMP in a concentration-dependent manner with an equilibrium disassociation constant (*K*_*d*_) of 724 nM and a binding stoichiometry of 2:1 (c-di-GMP to CC3689) (Fig. 1A). To test binding specificity, we performed UV cross-linking assays (5). His-CC3689 binding to ^33^P-labeled c-di-GMP was effectively outcompeted by the addition of unlabeled c-di-GMP but not by the addition of other nucleotides (Fig. 1B). From this, we concluded that CC3689 is a *bona fide* c-di-GMP binding protein.

### HfsK and its paralogs contribute to *C. crescentus* surface attachment by modifying the holdfast adhesin.

*C. crescentus* encodes two additional paralogs of *cc3689* (*cc2278* and *cc1244*) with sequence identities of around 30% (see Fig. S1A and B in the supplemental material). While the neighboring genes gave no hints about a possible function of *cc3689*, *cc2278* lies in an operon containing a gene for a predicted glycosyltransferase that is required for holdfast synthesis (27). In addition, when analyzing the genomic context of *cc3689* orthologs, we found that many of them cluster with genes predicted to function in EPS biogenesis (see Fig. S1C). Strains encoding such orthologs belong to different phyla, indicating that the connection of this protein class with EPS synthesis is of ancient evolutionary origin. Interestingly, in three closely related marine species, namely, *Maricaulis maris*, *Oceanicaulis alexandrii*, and *Woodsholea maritima* (39), orthologs of *cc3689* cluster with homologs of *C. crescentus* holdfast synthesis genes (40) (see Fig. S1D). On the basis of these observations and the results presented below, we renamed CC3689 holdfast synthesis protein K (HfsK).

10.1128/mBio.00294-17.5 FIG S1  Many HfsK orthologs are encoded in EPS synthesis clusters. (A) Multiple-sequence alignment of HfsK with its paralogs. Arginines mutated in this study are indicated by a square, and those shown to be involved in c-di-GMP binding are highlighted in red. Fully conserved residues are indicated by a star, and strong and weak conservation are indicated by two dots and one dot, respectively. (B) Sequence identities between the different HfsK paralogs (C) phylogenetic tree composed of the TOP58 hits of HfsK orthologs search with Blast (M. Johnson, I. Zaretskaya, Y. Raytselis, Y. Merezhuk, S. McGinnis, and T. L. Madden, Nucleic Acids Res **36:**W5–W9, 2008, doi: 10.1093/nar/gkn201). Colors indicate species that have orthologous genes in a conserved holdfast synthesis cluster (red) or in clusters with genes that do not show a connection with polysaccharide synthesis (green). The different corresponding phyla are indicated. (D) Schematic representation of conserved holdfast synthesis clusters that contain a gene orthologous to *hfsK* in comparison to the holdfast synthesis cluster of *C. crescentus*. Note that the holdfast synthesis-associated HfsK ortholog in *M. maris* is not under TOP58 Blast hits. gh, glycosylhydrolase; gt., glycosyltransferase. Download FIG S1, TIF file, 1 MB. Copyright © 2017 Sprecher et al. 2017 Sprecher et al. This content is distributed under the terms of the Creative Commons Attribution 4.0 International license.

**FIG 2:**
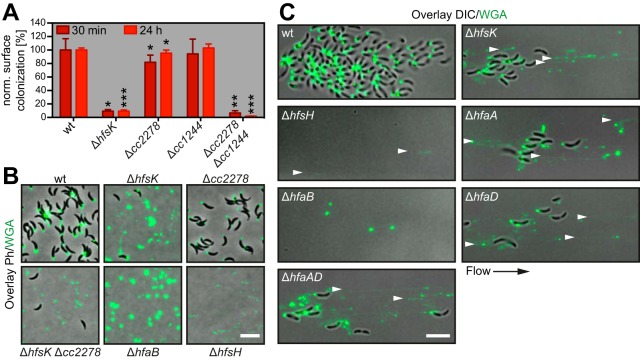
HfsK deletion leads to an incoherent holdfast that does not support surface colonization. (A) Surface colonization by the wild type (wt) and mutants lacking the *hfsK* paralog family determined by crystal violet staining of adherent cells after 30 min (dark red bars) and 24 h (light red bars) of growth in microtiter plates. norm, normalized per condition. Error bars show standard deviations (SD) of three independent experiments. *, **, and *** represent *P* values of <0.1, <0.01, and <0.001, respectively. (B) Analysis of holdfast shedding in *hfsK* and *cc2278* mutants, as well as in strains that shed (Δ*hfaB*) or form incoherent (Δ*hfsH*) holdfast. Shown are overlays of phase-contrast (Ph) and fluorescence images of adhered WGA-stained holdfast on glass coverslips after 2 h of cell adsorption. (C) Holdfasts analyzed under shear stress after 15 h of growth in a microfluidic channel with a constant flow of fresh medium containing OG-WGA. Shown is an overlay of fluorescence and inverted differential interference contrast (DIC) images. Arrowheads point to holdfast filaments. Scale bars in panels B and C, 5 μm.

To test whether *hfsK* and its paralogs *cc2278* and *cc1244* are involved in holdfast biogenesis, we engineered deletions of all three genes in the chromosome of *C. crescentus* wild-type strain CB15 and investigated surface colonization by the resulting mutant strains as a proxy for their ability to form a functional holdfast. The Δ*hfsK* mutant showed a 90% reduction in surface colonization after 30 min and after 24 h of growth compared to the wild type. In contrast, the Δ*cc2278* mutant showed only minor defects in surface colonization during the initial phase of growth (Fig. 2A; see Fig. S2A). Surface colonization was fully restored when the *hfsK* and *cc2278* mutants were complemented with a wild-type copy of the respective gene in *trans*, but the two proteins failed to cross-complement each other (see Fig. S2B). A Δ*hfsK* Δ*cc2278* double mutant showed lower surface colonization than the *hfsK* single mutant, indicating additive contributions of both proteins to surface attachment (Fig. 2A). Finally, deletion of the third paralog, *cc1244*, revealed no obvious phenotype alone or in combination with deletions of *hfsK* or *cc2278*. However, the Δ*hfsK* Δ*cc2278* Δ*cc1244* triple mutant failed to adhere completely (Fig. 2A; see Fig. S2A).

10.1128/mBio.00294-17.6 FIG S2  Attachment defect of *hfsK* mutants is caused by a less adhesive holdfast. (A, B) Surface colonization determined by crystal violet staining after 30 min (dark red bars) or 24 h (light red bars) of growth in microtiter plates. Values are normalized per condition. Comparison of the *hfsK* paralog family with strains that shed (Δ*hfaB*) and form incoherent (Δ*hfsH*) holdfast and the nonadherent *C. crescentus* NA1000 strain (A). Complementation and cross-complementation of the surface colonization defect of Δ*hfsK* and Δ*cc2278* mutant cells harboring a plasmid-borne, xylose promoter (P_*xyl*_)-driven copy of *hfsK* or *cc2278* or the empty vector (e.v.) alone (B). (C) Analysis of holdfast shedding. The fraction of ST cells from a liquid culture with visible OG-WGA-stained holdfast was counted (dark red bars). As a comparison, the fluorescence intensity of WGA-stained holdfast adhering to glass coverslips after 2 h of cell adsorption (as shown in Fig. 2B and panel D) was quantified (light red bars). (D) Representative images of OG-WGA-stained holdfast adhering to glass coverslips after 2 h of cell adsorption. Shown are overlays of phase-contrast (Ph) and fluorescence images. Scale bar, 5 μm. (E, F) Assessment of the involvement of HfsK and its paralogs in other c-di-GMP-controlled pathways. Quantification of colony size indicative of swimming after 3 days of growth on semisolid agar plates (E). Phage susceptibility was tested by adding serial dilutions of φCBK (pilus specific) and φCR30 (S-layer specific) phage lysate onto a lawn of cells (F). (G) Schematic view of an SCFS with FluidFM. The courses of pressure (p) and force (f) over time are plotted. The SCFS procedure and characteristic cantilever bending are shown. (H) Relative adhesion forces of wild-type (wt) and Δ*hfsK* mutant cells grown in PYE medium. Three to five cells per culture were measured as described in Text S1, and different glass substrates were investigated. For all conditions, the substrate contact times range from 0.5 to 6 h. Error bars show the SD of three independent cultures. (I) Comparison of the detachment breaking point of Δ*hfsK* mutant cells treated and measured as described for panel H. Cells were categorized according to their holdfast intensity, i.e., reversibly attached to glass without a visible OG-WGA-stained holdfast (no) or attached via a weakly or strongly fluorescently labeled holdfast indicative of short (minutes) and long (hours) contact times. (J) Coattachment of holdfast-null Δ*hfsJ* mutant strains with and without an intact *hfsK* and the Δ*hfaB* holdfast-shedding anchor mutant. The holdfast-null and shedding strains harbor a plasmid expressing eGFP and mCherry from P_*xyl*_, respectively. The strains were mixed 1:1 in PYE supplemented with 0.1% xylose and for 2 h allowed to adsorb to glass slides that were then stained with Alexa Fluor 350-WGA and washed before microscopy. Scale bar, 5 μm. In panels A to D, the error bars represent the SD of at least three independent experiments. *, **, and ***, represent *P* values of <0. 1, <0.01, and <0.001, respectively. ns, not significant. Download FIG S2, TIF file, 1.6 MB. Copyright © 2017 Sprecher et al. 2017 Sprecher et al. This content is distributed under the terms of the Creative Commons Attribution 4.0 International license.

In line with the strong surface colonization defect, the Δ*hfsK* mutant showed a severe reduction in holdfast biogenesis. Upon staining of holdfast with the Oregon green-labeled lectin wheat germ agglutinin (OG-WGA), 63% of the wild-type ST cells carried a holdfast while only 4% of the Δ*hfsK* mutant cells were holdfast positive (see Fig. S2C). Whereas strains carrying deletions of *cc2278* or *cc1244* showed normal holdfast formation, holdfast formation was completely abolished in a triple mutant lacking HfsK and its paralogs.

The observed reduction of Δ*hfsK* mutant cells bearing an adhesive holdfast could be explained by either diminished holdfast production or defective anchoring and increased shedding of holdfast material (28). To distinguish between these possibilities, wild-type and mutant strains were allowed to adsorb to glass surfaces for 2 h before the glass was washed, stained with OG-WGA, and analyzed by fluorescence microscopy. Glass surfaces incubated with the wild-type strain were covered with cells adhering via their holdfasts. In contrast, only a few cells of the Δ*hfsK* mutant remained attached after washing, yet the amount of holdfast material observed was comparable to that of the wild type (Fig. 2B; see Fig. S2C). Of note, this is similar to the shedding phenotype observed in a strain lacking the holdfast anchor protein HfaB (25, 28). Cells lacking CC2278 did not shed their holdfast but produced smaller, less intensely stained adhesins. A Δ*hfsK* Δ*cc2278* double mutant shed small holdfasts, again indicating that the two proteins affect holdfast properties independently. Intriguingly, the Δ*cc2278* Δ*cc1244* double mutant and the triple mutant completely failed to adhere to glass (see Fig. S2C and D). This and the observation that surface colonization of the Δ*cc2278* Δ*cc1244* double mutant was barely affected in polystyrene microtiter plates (see Fig. S2A) indicated that distinct members of this family of proteins might optimize the attachment to different surface chemistry.

Mutants with mutations in *hfsK* or its paralogs retained normal surface-adherent pili and active flagellar motors (see Fig. S2E and F), two c-di-GMP-dependent cellular appendages that are required for optimal surface attachment (22, 27, 29, 41). On the basis of these data, we propose that the reduction in surface colonization observed in *hfsK*, *cc2278*, and *cc1244* mutants can be attributed to defective holdfast biogenesis or, in the case of an *hfsK* mutant, possibly defective holdfast anchoring.

### HfsK contributes to holdfast cohesion and adhesion strength.

Recently, Wan and colleagues showed that a mutant lacking the polysaccharide deacetylase HfsH sheds holdfast material that is less cohesive and forms fiber-like structures when exposed to shear forces (19). The authors suggested that the degree of acetylation might be critical for the physical properties of holdfast. Similarly, the *N*-acyltransferase HfsK might influence the acetylation state of holdfast. To analyze holdfast performance under shear stress, wild-type and mutant bacteria were grown in a microfluidic device under permanent flow of fresh medium. After overnight growth, individual wild-type cells formed microcolonies with discrete foci of WGA-stained holdfast material at the adherent cell poles (Fig. 2C). Mutants lacking HfaB or HfsH were unable to attach but shed compact holdfast structures and faint holdfast fibers, respectively. In contrast, the *hfsK* mutant formed microcolonies smaller than those of the wild type with fluorescent trails of abraded, filamentous holdfast structures. Holdfast trails were generated by mutant cells that secreted holdfast material onto the surface while slowly drifting with the medium flow (see Movies S1 and S2). Some holdfast structures elongated into extended filaments from which cells were dangling for some time before the connection ruptured. Upon rupturing, several holdfast filaments bounced back like a released rubber band (see Movie S3), indicating that the cohesive and elastic properties of the holdfast are severely altered in the Δ*hfsK* mutant.

10.1128/mBio.00294-17.1 MOVIE S1  Holdfasts of *hfsK*-deficient cells are elongated to filaments under flow conditions. This is a time-lapse video of Δ*hfsK* mutant cells grown in a microfluidic channel in PYE supplemented with 1 μg/ml OG-WGA. Scale bar, 3 μm. Download MOVIE S1, AVI file, 0.8 MB. Copyright © 2017 Sprecher et al. 2017 Sprecher et al. This content is distributed under the terms of the Creative Commons Attribution 4.0 International license.

10.1128/mBio.00294-17.2 MOVIE S2  Cells deficient in *hfsK* can glide on surfaces. The arrow indicates a cell that is not able to make surface contact and is immediately flushed away. This is a time-lapse video of Δ*hfsK* mutant cells grown in a microfluidic channel in PYE supplemented with 1 μg/ml OG-WGA. Scale bar, 3 μm. Download MOVIE S2, AVI file, 2.5 MB. Copyright © 2017 Sprecher et al. 2017 Sprecher et al. This content is distributed under the terms of the Creative Commons Attribution 4.0 International license.

10.1128/mBio.00294-17.3 MOVIE S3  Holdfast of *hfsK*-deficient cells is elastic. The white arrow indicates a cell that dangles on a holdfast filament and shows the condensed holdfast after filament rupture. This is a time-lapse video of Δ*hfsK* mutant cells grown in a microfluidic channel in PYE supplemented with 1 μg/ml OG-WGA. Scale bar, 3 μm. Download MOVIE S3, AVI file, 1.7 MB. Copyright © 2017 Sprecher et al. 2017 Sprecher et al. This content is distributed under the terms of the Creative Commons Attribution 4.0 International license.

To analyze the adhesive forces of wild-type and mutant holdfasts more precisely, we used a single-cell force spectroscopy (SCFS) approach and fluidic force microscopy (FluidFM) technology. This setup enables single-cell manipulation by combining the precise force control of an atomic force microscope with a microfluidic device (42, 43) (see Fig. S2G). Comparison of detachment forces revealed that, on average, wild-type cells showed approximately five times stronger adherence than Δ*hfsK* mutant cells (see Fig. S2H). Of note, in several cases, cells could not be detached at all and were not included in the analysis. During holdfast biogenesis, the dimensions of the secreted structures increase over time (44). Thus, we next compared the adherence of Δ*hfsK* mutant cells with that of weaker (young holdfast) and more intense holdfast staining (mature holdfast). Larger holdfasts were indeed more likely to remain surface bound, with ruptures often occurring between the adhesin and the cell body (see Fig. S2I). These results indicated that holdfast from the Δ*hfsK* mutant, although more fragile and less cohesive, can still gain adhesion strength over time, as observed in holdfasts of wild-type cells (20).

The above-described experiments argued that holdfasts of Δ*hfsK* mutant cells, similar to those of Δ*hfsH* mutant cells, show altered cohesive or adhesive properties. However, it is unclear if the observed changes influence the overall properties of the holdfast material or its anchoring in the cell envelope. In line with the latter, microfluidic experiments exposed similar phenotypes of Δ*hfsK* mutant cells and mutants lacking the holdfast anchor protein HfaA or HfaD. When growing Δ*hfaA* mutants, Δ*hfaD* mutants, or double mutants in microfluidic devices, trails of WGA-stained material were observed, similar to the structures formed by Δ*hfsK* mutants (Fig. 2C). Thus, we asked whether the holdfast anchoring process is still functional in Δ*hfsK* mutant cells. For this, we took advantage of the observation that *hfs*, but not *hfa*, mutants, are able adhere to holdfasts shed by anchor mutants (21). We combined the Δ*hfsK* deletion with a Δ*hfsJ* deletion, which completely abolishes holdfast EPS formation (26), and tested the coattachment of these cells with a Δ*hfaB* mutant strain. Deletion of *hfsK* did not change the coattachment capacity of the holdfast-deficient strain (see Fig. S2J), suggesting that this strain produces an intact holdfast anchor.

Together, these results demonstrate that HfsK contributes to the effective surface adherence of *C. crescentus* by modulating the cohesive properties of the holdfast material and/or by facilitating the efficient anchoring of the adhesin in the cell envelope that is necessary to withstand strong shear forces.

### c-di-GMP controls HfsK compartmentalization.

Holdfast production is controlled by c-di-GMP and coincides with an upshift of c-di-GMP levels during the SW-to-ST-cell transition (27, 29). From this and from the observation that HfsK binds c-di-GMP, we anticipated that the activity of this protein might be controlled by c-di-GMP during the cell cycle, akin to other c-di-GMP effector proteins (30, 45, 46). Similar to the expression of other *hfs* genes (26), that of *hfsK* is specific to the late predivisional stage of the cell cycle (47). However, this does not result in significant changes in HfsK protein levels during the cell cycle (see Fig. S3A). Accordingly, HfsK levels showed only minor changes in engineered strains with different c-di-GMP levels (see Fig. S3B and C). Thus, c-di-GMP affects HfsK abundance only marginally, making it unlikely that holdfast maturation is controlled by c-di-GMP at the level of HfsK expression or stability.

10.1128/mBio.00294-17.7 FIG S3  Levels of HfsK do not change during the cell cycle. (A) HfsK protein levels of synchronized wild-type (wt) cells grown in M2G through one cell cycle analyzed by immunoblot assay probed with anti-HfsK antibodies. Cell cycle-regulated protein CtrA is shown as a control. (B) Measurement of c-di-GMP concentrations of wild-type cells, cells that have no c-di-GMP metabolism (rcdG0), and cells with elevated c-di-GMP levels (rcdG0::*dgcZ*). Expression of *dgcZ* was induced by the addition of 0.5 mM IPTG. Error bars show the SD of three technical replicates. (C) Impact of c-di-GMP on HfsK homeostasis. HfsK protein levels analyzed by immunoblot assay in wild-type, rcdG0, and rcdG0::*dgcZ* (induced with 0.5 mM IPTG) cells. ClpX was used as a loading control. Quantification of immunoblot assays of four independent experiments is shown at the bottom. Error bars show the SD. * and ** represent *P* values of <0.1 and <0.01, respectively. Download FIG S3, TIF file, 1 MB. Copyright © 2017 Sprecher et al. 2017 Sprecher et al. This content is distributed under the terms of the Creative Commons Attribution 4.0 International license.

**FIG 3:**
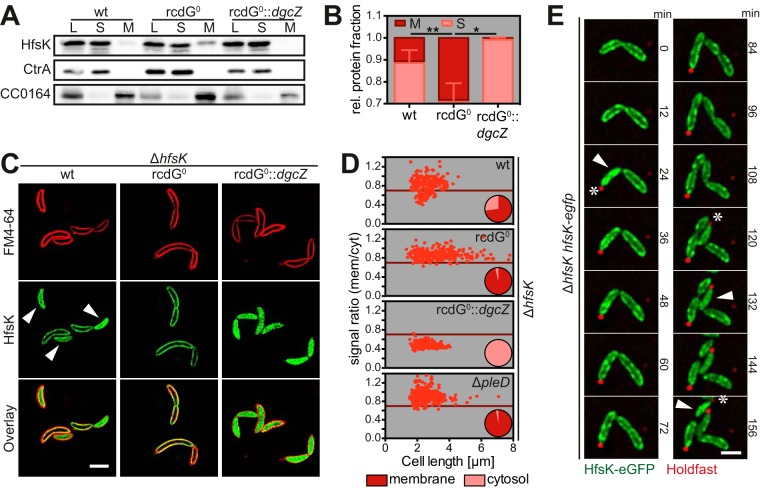
c-di-GMP controls HfsK localization. (A, B) HfsK localization in wild-type (wt), rcdG0 (c-di-GMP low), and rcdG0::*dgcZ* (c-di-GMP high) cells analyzed by cell fractionation, followed by immunoblotting. CtrA (cytosolic) and CC0164 (inner membrane) were used as controls. (A) Lanes: cell lysates, L; soluble fraction, S; membrane fraction, M. (B) Quantification of three independent cell fractionations showing the fractions of soluble (light red) and membrane-associated (dark red) HfsK. Error bars show the SD, * and ** represent *P* values of <0.1 and <0.01, respectively. (C) 3D-SIM images of HfsK-eGFP in cells with different c-di-GMP levels. Staining was done with the membrane dye FM4-64. Arrowheads indicate cells with cytosolic HfsK-eGFP. Scale bar, 2 µm. (D) Quantification of HfsK-eGFP localization in standard fluorescence microscopy images. The ratio of the average signal intensities in the membrane and cytosolic compartments is correlated with cell length. The pie chart inset shows the fraction of cells with a membrane-associated (ratio_membrane-cytosol_, > 0.7; dark red) or cytosolic (ratio, ≤0.7; light red) GFP signal. *n* = 250 cells per strain in two independent experiments. (E) Time-lapse video of Δ*hfsK* P_*van*_-*hfsK-egfp* mutant cells grown on PYE agarose pads supplemented with rhodamine-WGA and vanillic acid at 30°C. Cells with dispersed HfsK-eGFP (arrowheads) and the first appearance of holdfast in each cell (asterisks) are indicated. Scale bar, 1 µm. In all of the images, expression of *egfp* constructs and *dgcZ* was induced with 0.55 mM vanillic acid and 0.5 mM IPTG, respectively.

Although assembly and maturation of EPS generally occur in or at the cytoplasmic membrane (48), HfsK is predicted to be a cytosolic protein (49). We used cell fractionation experiments to determine HfsK localization. After the ultracentrifugation of cell lysates, about 90% of the HfsK remained in the soluble fraction, while 10% was retained in the pellet (Fig. 3A and B). This indicated that HfsK is at least partially membrane associated, possibly by binding to an interaction partner in the membrane. In agreement with this idea, HfsK was lost from the membrane fraction when lysates were treated with increasing salt concentrations that are known to disturb such interactions (50) (see Fig. S4A). Importantly, none of the known inner membrane components of the holdfast synthesis machinery was required for sequestration of HfsK to the membrane (see Fig. S4B).

10.1128/mBio.00294-17.8 FIG S4  Membrane association of HfsK depends on electrostatic interaction but not with the holdfast synthesis machinery. (A) HfsK localization analyzed by cell fractionation followed by immunoblotting. The cytosolic protein CtrA and the inner membrane protein CC0164 were used as controls. Lanes: cell lysates, L; soluble fraction, S; membrane fraction, M. Wild-type cell lysates were supplemented with increasing concentrations of NaCl to disrupt potential electrostatic interactions. (B) Comparison of HfsK-eGFP localization in Δ*hfsK* mutant cells and Δ*hfsK* mutant cells lacking the entire holdfast synthesis machinery (Δ*hfsABCDEFGHIJ*) grown in the presence of 0.55 mM vanillic acid. Localization quantification was done as described in the legend to Fig. 3D. At least 720 cells per strain from two independent experiments were analyzed. (C) HfsK localization analyzed by cell fractionation followed by immunoblotting with the same controls as in panel A. c-di-GMP concentrations were enhanced in the rcdG0 strain by supplementing all buffers with 10 µM c-di-GMP or by additionally expressing *dgcZ* from P_*lac*_ by the addition of 0.5 mM IPTG in addition to c-di-GMP supplementation of buffers. (D) Immunoblot assay of all of the HfsK-eGFP fusion constructs used in this study probed with anti-GFP and anti-HfsK antibodies. Degradation bands correspond to a truncation within the eGFP protein that leads to complete fluorescent signal loss (H.-K. Kim and B.-K. Kaang, Brain Res Bull **47:**35–41, 1998, doi: 10.1016/S0361-9230(98)00020-3) and thus does not impact microscopy analysis. (E) Functionality of mCherry fusions compared to that of a wild-type (wt) strain harboring the control empty vector (e.v.) in a surface colonization assay scored after 24 h of growth in microtiter plates in the presence of 0.1% xylose. Error bars represent the SD of three independent experiments. * and *** represent *P* values of <0.1 and <0.001, respectively, calculated with an unpaired *t* test. (F) Localization of early holdfast synthesis proteins compared to that of HfsK, all fused to mCherry and expressed from P_*xyl*_ by the addition of 0.1% xylose in the respective deletion background. Representative fluorescence microscopy images are shown. Scale bar, 10 µm. (G) Ratio of average membrane and cytosol signals. eGFP expressed from P_*xyl*_ in wild-type cells with 0.1% xylose was used as a cytosolic marker, and the membrane dye FM4-64 was used as a membrane marker. Pie charts show that, with an arbitrarily set threshold of 0.7, 100% of all GFP and FM4-64 signals are categorized as cytosolic and membrane associated, respectively. (H) Localization of HfsK-eGFP in Δ*hfsK* P_*van*_-*hfsK-egfp* mutant cells grown on PYE agarose pads containing 0.55 mM vanillic acid. Newborn swarmer cells (cell division, 0 min) were followed through one cell cycle. Localization in 32 cells originating from two independent experiments was quantified as described in the legend to Fig. 3D. Download FIG S4, TIF file, 0.9 MB. Copyright © 2017 Sprecher et al. 2017 Sprecher et al. This content is distributed under the terms of the Creative Commons Attribution 4.0 International license.

To test if the membrane association of HfsK is c-di-GMP controlled, cell fractionation was carried out with a newly constructed strain that lacks all of the genes encoding DGCs and PGEs (rcdG0) and with the same strain harboring a P_*lac*_-driven copy of *dgcZ* from *E. coli* (rcdG0::*dgcZ*), which allows tuning of intracellular c-di-GMP levels. Expression of the DgcZ DGC (51) in this background produced c-di-GMP levels 6-fold higher than those of the wild type (see Fig. S3B). In the rcdG0 strain, the fraction of membrane-associated HfsK increased to about 30%, while the rcdG0::*dgcZ* strain had lost the HfsK protein from the membrane fraction almost entirely (Fig. 3A and B). Likewise, when c-di-GMP was added to cell extracts of the rcdG0 strain before fractionation, HfsK primarily localized to the cytosol (see Fig. S4C). These results indicated that c-di-GMP modulates HfsK membrane interaction, with high levels of c-di-GMP promoting its cytosolic state and low levels of c-di-GMP stimulating its association with the membrane.

### HfsK dynamically repositions to the cytoplasm during the cell cycle.

To more carefully analyze HfsK localization and its association with the cytoplasmic membrane, we expressed a P_*van*_-driven chromosomal copy of *hfsK-eGFP* in the Δ*hfsK* mutant strain (see Fig. S4D). Localization of HfsK-enhanced green fluorescent protein (eGFP) relative to the membrane was visualized by superresolution three-dimensional structured illumination microscopy (3D-SIM) after cells were stained with the membrane-specific dye FM4-64. While the majority of cells showed peripheral HfsK-eGFP colocalizing with the membrane stain, HfsK-eGFP was dispersed in the cytosol in a subfraction of the population (Fig. 3C and D). Of note, with the exception of HfsH, which was reported to be cytosolic (19), all functional mCherry fusions of proteins catalyzing early steps of holdfast biogenesis (see Fig. S4E) localized to the membrane but lacked the cytosolic subfraction characteristic of HfsK (see Fig. S4F).

To investigate if these changes are driven by c-di-GMP, HfsK-eGFP localization was analyzed in cells harboring different c-di-GMP levels. Strikingly, in the rcdG0 strain, HfsK-eGFP showed strong membrane localization in a large majority of the cells, while HfsK-eGFP was entirely cytosolic in the rcdG0::*dgcZ* strain (Fig. 3C and D). Note that the rcdG0 strain constructed in this study shows a filamentous morphology similar to that of the cdG0 strain lacking all DGCs (29). To carefully quantify HfsK-eGFP localization at the single-cell level, averages of the fluorescent signals at the cell periphery and in the cytosol were determined and ratios were calibrated by using soluble eGFP and the membrane dye FM4-64 (see Fig. S4G). The fraction of cells with membrane-associated HfsK-eGFP ranged from 0% in the rcdG0::*dgcZ* strain to roughly 70% in the wild-type background and 96% in the rcdG0 background (Fig. 3D). Importantly, wild-type cells with membrane-associated HfsK-eGFP included the entire spectrum of measured cell length, while cells with a cytosolic signal were all short. This suggested that HfsK distribution changes during the cell cycle. To test this, time-lapse experiments were carried out with a Δ*hfsK* mutant strain expressing HfsK-eGFP. HfsK-eGFP was membrane associated in newborn SW cells but became cytosolic about 24 min after division and shortly after the appearance of holdfast (Fig. 3E; see Fig. S4H). About 12 min after its dispersal, HfsK-eGFP gradually relocalized to the membrane, coincident with cells starting to elongate and divide. These observations indicated that HfsK transiently delocalizes in newly differentiated ST cells, coincident with peak c-di-GMP levels during the cell cycle. In line with this idea, HfsK-eGFP was not discharged from the membrane fraction in a strain lacking PleD, the main DGC responsible for the upshift of c-di-GMP during the SW-to-ST transition (29, 52) (Fig. 3D).

Together, these results demonstrated that HfsK localization is dynamic and indicated that its repositioning to the cytoplasm during the SW-to-ST transition is driven by peak levels of c-di-GMP.

### The C terminus is required for the activity and membrane localization of HfsK.

**FIG 4:**
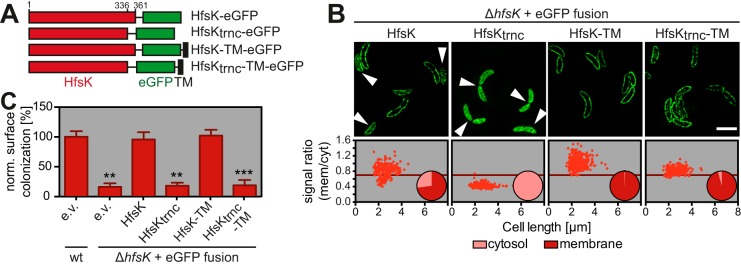
The C terminus of HfsK is an important determinant of its localization and function. (A) Schematic representation of the HfsK-eGFP mutants used in this experiment. Amino acid positions are indicated at the top. The drawing is not to scale. TM, transmembrane domain of SecE. (B) Localization quantification of different HfsK-eGFP mutants expressed by the addition of 0.55 mM vanillic acid as described in the legend to Fig. 3D. *n* = 260 cells per strain from two independent experiments. Representative 3D-SIM images for visualization are shown at the top. Arrowheads indicate cells with dispersed HfsK-eGFP. Note that because of the weak signal intensity of HfsK_trnc_-TM, the 3D-SIM image recreation resulted in images with a high background signal level. Scale bar, 3 µm. (C) Functionality of HfsK-eGFP mutants compared to that of a wild-type strain harboring the control empty vector (e.v.) in surface colonization after 24 h of growth in microtiter plates in the presence of 0.1 mM vanillic acid. Error bars show the SD of three independent experiments. ** and *** represent *P* values of <0.01 and <0.001, respectively.

Despite low sequence homology, GNAT proteins have a remarkably conserved core fold (38). The closest homolog of HfsK with a solved 3D structure is FemX, an enzyme involved in peptidoglycan synthesis in Gram-positive bacteria (53, 54). Both proteins have two GNAT domains in tandem. Sequence comparisons revealed that HfsK has a short C-terminal extension of 25 amino acids that is absent in FemX (see Fig. S5A). Surprisingly, an eGFP fusion protein with truncated HfsK (HfsK_trnc_) that lacks this extension, although being stable, lost its characteristic membrane association and, in contrast to full-length HfsK-eGFP, failed to complement the Δ*hfsK* phenotype (Fig. 4A to C; see Fig. S4C). HfsK and HfsK_trnc_ localized exclusively to the membrane when fused to the transmembrane (TM) helix of *C. crescentus* SecE, which is sufficient to restrict reporter proteins to the membrane (55) (Fig. 4A and B). However, these proteins harboring the TM from SecE failed to respond to changes in c-di-GMP (see Fig. S5B). Whereas TM-tagged wild-type HfsK was fully functional, cells expressing HfsK_trnc_ did not support surface colonization even if shuttled to the cytoplasmic membrane by the exogenous TM segment (Fig. 4C). Thus, the C terminus of HfsK is required for its membrane localization and for its function. These experiments also indicated that membrane-associated HfsK represents the active species of the protein and that release of HfsK from the membrane at high levels of c-di-GMP might lead to its inactivation.

10.1128/mBio.00294-17.9 FIG S5  HfsK localization depends on its C terminus. (A) Alignment of HfsK with FemX of *Weissella viridescens* based on structure prediction. The quality of conservation is indicated as follows: low, -; neutral, ⋅ ; high, +; very high, |. The C-terminal amino acids that were deleted in the HfsK_trnc_ mutant are highlighted by the red line. (B) Localization of P_*van*_-HfsK-eGFP mutant proteins expressed as the sole copy of HfsK in the wild-type, rcdG0, and rcdG0 *dgcZ* (plus 0.5 mM IPTG) backgrounds by the addition of 0.55 mM vanillic acid. Representative 3D-SIM images are shown at the top. The bottom shows localization quantification as described in the legend to Fig. 3D. At least 255 cells per strain from two independent experiments were analyzed. Scale bar, 3 μm. (C) Quantification of fluorescence intensity of adhered rhodamine-WGA-stained holdfast of R mutant strains on glass coverslips after 2 h of adsorption. Error bars show the SD of three independent experiments. Download FIG S5, TIF file, 1.9 MB. Copyright © 2017 Sprecher et al. 2017 Sprecher et al. This content is distributed under the terms of the Creative Commons Attribution 4.0 International license.

### c-di-GMP drives HfsK to the cytosol but is dispensable for its activity.

**FIG 5:**
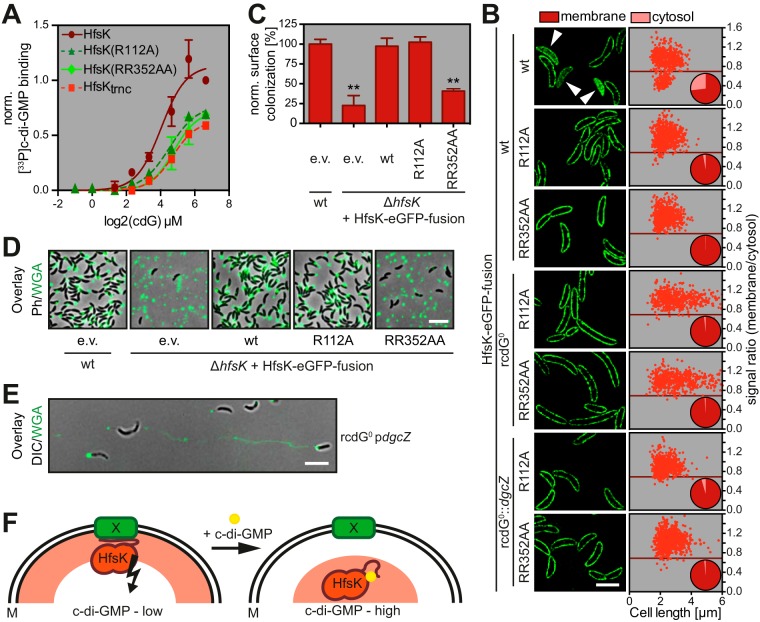
c-di-GMP binding is required for HfsK delocalization but not for HfsK-mediated surface colonization. (A) Quantified autoradiographs of 1 μM purified StrepII-HfsK mutants that were UV cross-linked to increasing concentrations of [^33^P]c-di-GMP. (B) Localization analysis of different arginine mutants fused to GFP expressed by the addition of 0.55 mM vanillic acid. Representative 3D-SIM images for visualization (left) and localization quantification, as described in the legend to Fig. 3D, are shown. *n* = 575 cells per strain from two independent experiments. Expression of *dgcZ* was induced with 0.5 mM IPTG. Scale bar, 3 µm. (C) Functionality of HfsK arginine mutants compared to that of a wild-type (wt) strain harboring the control empty vector (e.v.) in surface colonization after 24 h of growth in microtiter plates in the presence of 0.1 mM vanillic acid. (D) Analysis of adhered OG-WGA-stained holdfast on coverslips after 2 h of cell adsorption. Representative overlays of fluorescence and phase-contrast (Ph) images are shown. Scale bar, 5 μm. (E) Holdfasts of a strain with high c-di-GMP levels analyzed under shear stress after 15 h of growth in a microfluidic channel with a constant flow of fresh medium containing OG-WGA. Shown is an overlay of fluorescence and inverted differential interference contrast (DIC) images. Scale bar, 5 μm. (F) Model of HfsK regulation. General protein localization is represented by red shades. Active HfsK binds to membrane component X in the absence of c-di-GMP via its C terminus. Upon c-di-GMP binding, the C terminus rearranges to accommodate the nucleotide and the protein is inactivated and disperses in the cytosol. M, membrane. The error bars in panels A and B show the SD of three independent experiments. **, *P* < 0.01.

We have shown above that both c-di-GMP and the C terminus of HfsK are important for the localization of the protein during the cell cycle. This indicated that the C terminus of HfsK itself could be targeted by c-di-GMP and could contribute to c-di-GMP binding. In agreement with this, we found that a StrepII-HfsK fusion lacking the 25 C-terminal amino acids (HfsK_trnc_) bound c-di-GMP more weakly than wild-type HfsK did (Fig. 5A). Structural examination of c-di-GMP binding proteins had revealed important roles for arginine residues in ligand binding (56). We thus generated the mutant protein HfsK(RR352AA) that had two central arginine residues of the C terminus (see Fig. S1A) changed to alanine. Similar to shortened HfsK_trnc_, this mutation reduced the affinity for c-di-GMP (Fig. 5A). To identify residues located in the core region of HfsK that are involved in c-di-GMP binding, we compared the sequences of HfsK and its two *C. crescentus* paralogs. Because HfsK and CC1244, but not CC2278, were identified by CCMS, we searched for arginine residues that are conserved in HfsK and CC1244 but not in CC2278 (see Fig. S1A). Several of these residues of HfsK (R102, R112, R151, R240, and R267) were replaced with alanine, and the resulting mutant proteins were expressed as StrepII-tagged fusions in *E. coli*. Binding studies with radiolabeled c-di-GMP identified HfsK_R112a_ as the only mutant variant with reduced binding affinity for c-di-GMP (Fig. 5A). These results suggested that R112, as well as arginine residues located in the HfsK C terminus, may contribute to c-di-GMP binding.

Importantly, HfsK-eGFP fusions containing R112A or RR352AA point mutations remained membrane associated even in strains harboring high levels of c-di-GMP, arguing that they no longer respond to the second messenger *in vivo* (Fig. 5B). Functional analysis revealed that the HfsK_RR352AA_-eGFP fusion failed to restore the Δ*hfsK* phenotype, underlining the importance of the C terminus for protein function. In contrast, expression of the HfsK_R112a_-eGFP fusion was able to substitute for HfsK in surface colonization and holdfast biogenesis (Fig. 5C and D; see Fig. S5C). Together, these data support a model in which c-di-GMP binding determines HfsK subcellular localization and possibly HfsK activity. Our data are in line with the view that binding of c-di-GMP inactivates HfsK by sequestering the protein away from a membrane-associated active conformation. In agreement with this view, we found that unphysiologically high levels of c-di-GMP not only severely reduced surface colonization but, akin to the Δ*hfsK* mutant, changed the cohesive properties of holdfast structures (Fig. 5E; see Fig. S6A to C). The finding that the C terminus of HfsK is essential for its function and for its subcellular localization and contributes to c-di-GMP binding implies that this part of the protein is the central regulatory hub controlling HfsK dynamics and activity in response to the second messenger.

10.1128/mBio.00294-17.10 FIG S6  High c-di-GMP levels cause holdfast shedding and a severe surface colonization defect. (A) Surface colonization of a strain expressing *dgcZ* from a high-copy-number plasmid or harboring the empty vector (e.v.) control determined by crystal violet staining after 24 h of growth in the presence of 200 μM IPTG in microtiter plates. (B) Amount of shed holdfast quantified in fluorescence microscopy images of mid-log-phase cultures that were stained with OG-WGA and directly spotted onto agarose pads. The total number (n) of assigned holdfasts is indicated. (C) Analysis of OG-WGA-stained holdfast adhering to coverslips after 2 h of cell adsorption. Representative fluorescence and phase-contrast (Ph) images are shown. Scale bar, 5 μm. (D) Structural model of HfsK based on FemX (PDB ID 3GKR). Colors show the N-terminal GNAT domain (red) and the C-terminal GNAT domain (green). As the HfsK protein is longer than FemX, the C terminus appears unstructured in the model. The C-terminal helical stretches predicted separately by JPred (A. Drozdetskiy, C. Cole, J. Procter, and G. J. Barton, Nucleic Acids Res **43:**W389–W394, 2015, doi: 10.1093/nar/gkv332) are blue. Arginines involved in c-di-GMP binding are purple. The error bars in panels A and B represent the SD of three independent experiments. *, **, and *** represent *P* values of <0.1, <0.01, and <0.001, respectively. norm., normalized; wt, wild type. Download FIG S6, TIF file, 2.3 MB. Copyright © 2017 Sprecher et al. 2017 Sprecher et al. This content is distributed under the terms of the Creative Commons Attribution 4.0 International license.

## DISCUSSION

The second messenger c-di-GMP controls EPS production in a wide range of bacteria by stimulating the activity of glycosyltransferases directly or via adaptor proteins (15, 57–59). By this means, c-di-GMP directly affects the polymerization and rate of secretion of EPS across the inner membrane. Here we describe the first c-di-GMP effector that is not required to adjust the amount of EPS produced but rather controls EPS adhesin modification and thus changes its physical properties and strength. However, HfsK activity does not seem to depend on c-di-GMP. Rather, our data argue that c-di-GMP affects HfsK negatively, possibly to adjust or coordinate its activity with other processes of holdfast biogenesis. Because cells that lack c-di-GMP are unable to produce holdfast (29), additional c-di-GMP-controlled catalytic components must contribute to this process. This example nicely illustrates that c-di-GMP can influence EPS production both quantitatively and qualitatively.

Cells lacking HfsK produce normal amounts of holdfast material. However, mutant holdfasts form elastic and abrasive filaments that are unable to withstand strong shear forces, suggesting that they have reduced cohesiveness and stability. A similar change in the physical properties of the holdfast adhesin was described for mutants deficient in HfsH. HfsH was proposed to deacetylate holdfast EPS precursors and, by doing that, unmask amine groups that might serve as holdfast anchoring sites (19). This is consistent with our findings that cells lacking the holdfast anchoring protein HfaA or HfaD display comparable holdfast behavior under shear stress. In contrast to the Δ*hfaA* Δ*hfaD* double mutant, shed holdfasts of Δ*hfaB* mutant cells did not deform under shear stress and retained a globular shape. Because HfaA and HfaD are thought to be exported and inserted into the outer membrane by the action of HfaB, these factors should, in principle, behave epistatically. However, similar observations were made by Hardy and colleagues indicating that HfaB likely adopts additional roles (28). For instance, it is possible that HfaB directly contributes to EPS anchoring and that in its absence shedding of the holdfast is unrestrained. Altogether, the similarity of the mutant phenotypes suggested that the putative acyltransferase HfsK, the deacetylase HfsH, and the holdfast anchor proteins HfaD and HfaA may be part of the same pathway that is required for holdfast anchoring and proper holdfast cohesion—two aspects that seem to be interdependent.

The exact role of HfsK in this pathway remains unclear. The closest homologs with known structure are the Fem proteins of Gram-positive bacteria that transfer aminoacyl moieties to peptidoglycan sugar precursors (53, 54). Given the low overall sequence similarity, the functional versatility of GNAT proteins, and their diversity in terms of acyl donors and acceptors (38), it is difficult to make predictions about the catalytic role of HfsK. It might transfer an acyl group to amines exposed by the action of HfsH (19). Depending on the nature of this acyl group, it could be involved in the covalent linkage of polysaccharide moieties to anchor proteins (60) or participate in electrostatic interactions required for adhesion, cohesion, or anchoring. This is in line with the observation that isolated holdfasts from a Δ*hfsH* mutant showed reduced electrostatic interactions with the substrate (20). While we cannot fully exclude the possibility that HfsK acylates an anchor protein to provide cross-linking sites, several observations indicated that HfsK chemically modifies holdfast EPS precursors directly (1). HfsK homologs are genetically coupled to various EPS synthesis systems (2). Deletion of the paralogs *cc2278* and *cc1244* affects the adhesin without an observable shedding phenotype (3). Cells lacking HfsK are able to adhere to shed holdfasts of a Δ*hfaB* mutant, indicating an intact anchor mechanism (4). HfsK colocalizes with other holdfast components involved in EPS precursor biogenesis. This is in contrast to holdfast export and anchoring proteins that localize to the cell pole where holdfast is assembled (25, 28, 61). On the basis of these arguments, we propose that HfsK acylates the EPS component of the holdfast and that this modification is necessary for proper holdfast cohesion and anchoring.

HfsK was originally isolated by a c-di-GMP-specific capture method and was shown to specifically bind c-di-GMP *in vitro*. The binding affinity of HfsK lies in the submicromolar range, which correlates well with the peak concentrations of c-di-GMP during *C. crescentus* SW-to-ST differentiation (29, 31). These values are in line with our findings that the protein delocalizes in a c-di-GMP-dependent manner coincident with holdfast formation during the cell cycle. HfsK may thus be retained at the cytosolic membrane when c-di-GMP levels are low or intermediate and be transiently released from the membrane during a short period of the cell cycle, when c-di-GMP reaches a high concentration. Our data also suggest that the membrane-associated form of HfsK is catalytically active, while membrane release results in its inactivation. In line with this, we found that the R112A mutant c-di-GMP binding protein permanently localized to the membrane while retaining its activity for holdfast formation. Several observations point to the C terminus as a central determinant of HfsK localization and catalytic activity. Mutants lacking the C terminus failed to localize to the membrane and were inactive even when forced to bind to the membrane artificially. Moreover, the C terminus is also involved in c-di-GMP binding. A mutant protein lacking two central arginine residues within this region failed to efficiently bind c-di-GMP and remained membrane associated throughout the cell cycle irrespective of the c-di-GMP concentration. Unlike the R112A mutant protein, the RR352AA variant was inactive, indicating that this site may be the core of HfsK control. We propose a model in which the C terminus of HfsK serves as an interaction site for a putative membrane partner (Fig. 5F). In this model, membrane tethering is necessary for HfsK activity while c-di-GMP binding interferes with the tether and leads to delocalization and inactivation of HfsK. On the basis of our data, we envisage that the arginine residues in the C terminus are involved in the c-di-GMP binding and activity of HfsK, offering a simple switch through which c-di-GMP can control conformation, membrane association, and catalytic activity. A FemX-derived structure model of HfsK could provide a molecular frame for this c-di-GMP-mediated switch (see Fig. S6D). Residue R112, which is localized on the surface of one of the GNAT domains, and R352 and/or R353 in the C terminus might jointly contribute to c-di-GMP binding. Accordingly, ligand binding would provoke the C terminus to swing back and interact with the GNAT core. To clarify such mechanistic details, additional biochemical and structural studies with HfsK and c-di-GMP are needed.

This study represents one of few examples of a c-di-GMP effector protein that is inactivated by ligand binding (30, 62). It remains unclear why HfsK activity would need to be turned off during the cell cycle and why this process is linked to peak levels of c-di-GMP. Given the timing of HfsK delocalization, it is possible that it is involved in some early step of holdfast biogenesis, catalyzing a reaction that is detrimental for later steps of holdfast export or maturation. If so, *C. crescentus* may elegantly use c-di-GMP for dual control of holdfast biogenesis. During the SW-to-ST transition, when c-di-GMP levels begin to increase, one or several key enzymes may be turned on to initiate holdfast biogenesis, but when c-di-GMP levels peak, the cell might turn off an enzyme(s) that is no longer needed or damaging. Alternatively, HfsK may engage in additional processes. For several holdfast synthesis steps, redundant functional equivalents exist, with one copy being encoded in the *hfs* operons and its paralog(s) being encoded elsewhere. It was proposed that paralogs may act in other pathways but can contribute to holdfast synthesis because of substrate similarities (23). It is plausible that HfsK interferes with related cellular pathways required for capsule, lipopolysaccharide, O-antigen, or possibly even peptidoglycan synthesis. In this case, proper timing of enzyme activity during the cell cycle could help prevent substrate depletion or leakage, thereby providing a rationale for c-di-GMP-mediated control.

## MATERIALS AND METHODS

### Bacterial strains and growth conditions.

The bacterial strains and plasmids used in this study are described in Text S1. *E. coli* strains were grown at 37°C or 30°C in Luria broth, and *C. crescentus* strains were grown at 30°C in peptone yeast extract (PYE) or M2 minimal medium supplemented with 0.1% glucose (M2G) under aeration or on the respective 1.5% agar medium plates. If required, media were supplemented with the appropriate antibiotics (*E. coli*, 50/30 [solid/liquid in μg/ml] kanamycin and 30/20 chloramphenicol; *C. crescentus*, 20/5 kanamycin and 20/0 nalidixic acid); the inducer isopropyl-β-d-thiogalactopyranoside (IPTG) at 0.3 0.5, or 0.75 mM; xylose at 0.1%; and vanillic acid at 0.1 or 0.55 mM. If required, cell cultures were synchronized by density gradient centrifugation (63) (see Text S1).

10.1128/mBio.00294-17.4 TEXT S1  Materials and methods used in this study. Download TEXT S1, PDF file, 0.3 MB. Copyright © 2017 Sprecher et al. 2017 Sprecher et al. This content is distributed under the terms of the Creative Commons Attribution 4.0 International license.

### Attachment assay.

Overnight (24-h) or mid-log-phase (30-min) cultures were diluted 1:32 or to an optical density at 600 nm (OD_660_) of 0.3, respectively, and grown in 96-well polystyrene microtiter plates at 30°C under aeration for the times indicated. Plates were then rinsed thoroughly with water, incubated for 30 min with 0.1% (wt/vol) crystal violet–1% methanol–isopropanol, rinsed again, and dried, and the adherent crystal violet was dissolved in 20% acetic acid before absorption at 600 nm was measured (see Text S1).

### Fluorescence microscopy.

Bacteria in mid-log phase were mounted on 1% agarose pads containing water (snapshots) or PYE containing appropriate supplements (time-lapse videos). The specifications of the microscopes used can be found in Text S1. Images showing protein localization were deconvolved with softWoRx or Huygens software.

### Holdfast and membrane stain.

Membranes were visualized on 1% agarose pads containing 0.66 μg/ml FM4-64 dye (Molecular Probes, USA). To visualize holdfast, cultures were incubated with 1 μg/ml OG-WGA (Invitrogen, USA) before microscopy or 2.66 µg/ml tetramethylrhodamine-WGA was added to the agarose pads directly. Adherent holdfast visualization on glass was adapted from reference 25. Overnight cultures or, if required, preinduced mid-log-phase cultures were diluted to an OD_660_ of 0.15 and grown in polystyrene plates containing round 12-mm borosilicate coverslips for 2 h at 30°C under aeration. The coverslips were then stained for 15 min with 2.5 µg/ml WGA coupled to Oregon green, tetramethylrhodamine, or Alexa Fluor 350 (Invitrogen); rinsed with water; and mounted on 1% agarose pads. For quantification, the mean gray value was measured with the FIJI software (64), and the measured background fluorescence of the holdfast-minus NA1000 strain was subtracted (see Text S1).

### Quantification of protein localization.

Quantitative fluorescent signal measurements were performed with a MatLab-based program developed by our group (WHISIT; available from MathWorks, Natick, MA). WHISIT calculated the average pixel fluorescent signal intensity of the membrane and cytoplasmic compartments. The membrane compartment was defined to enclose the first four intracellular pixels flanking the cell outline that was computed by Oufti (65) on phase-contrast images, while the remaining intracellular pixels were defined as the cytoplasmic compartment (see Text S1).

### Microfluidics.

Mid-log-phase cells were used to fill polydimethylsiloxane-based microfluidic devices (66) consisting of a single channel 10 mm long, 40 μm wide, and 25 μm high before a constant flow (0.002 μl/s) of PYE medium supplemented with 1 μg/ml OG-WGA was installed to allow growth (see Text S1).

### Cell fractionation.

Cells were lysed in CellFrac buffer (phosphate-buffered saline, 1× cOmplete mini EDTA-free protease Inhibitor [Roche], 2.5 μg/ml DNase I [Roche]) with a French pressure cell and centrifuged to remove cell debris (10 min, 18,000 × *g*, 4°C). The cleared lysate was then centrifuged at high speed (1 h, 100,000 × *g*, 4°C) to separate soluble from insoluble proteins. The supernatant was removed and kept as the soluble fraction, whereas the pellet was washed and resuspended in the original volume of CellFrac buffer. The cleared lysate and soluble and pellet fractions were further analyzed by immunoblotting (see Text S1).

### Protein purification.

Proteins expressed from pET28aStrepII plasmids were purified from cleared lysates with Strep-Tactin Superflow plus resins (Qiagen) in accordance with the manufacturer’s protocol. HfsK-His was purified on a 5-ml HisTrap HP column (GE Healthcare), and the His-tagged protein was eluted with a gradient of elution buffer containing 20 mM Tris (pH 8.5), 0.5 M NaCl, 3 mM β-mercaptoethanol, 0.1%Tween 20, and 500 mM imidazole and concentrated on a Superdex 200 10/300 GL Increase gel filtration column (GE Healthcare) equilibrated with 30 mM HEPES (pH 7.4), 0.3 M NaCl, 3 mM β-mercaptoethanol, and 5 mM MgCl_2_ (see Text S1).

### ITC.

ITC measurements were performed with a VP-ITC isothermal titration calorimeter (MicroCal) with 13 μM HfsK in the cell and 211 μM c-di-GMP in the syringe (buffer: 30 mM HEPES [pH 7.4], 0.3 M NaCl, 5 mM MgCl_2_, and 3 mM β-mercaptoethanol) at 22°C. The first injection of 3 μl was followed by 29 injections of 10 μl. The data were analyzed with the MicroCal version of ORIGIN and fitted with the “One binding site model” of ORIGIN (see Text S1).

### UV cross-linking with [^33^P]c-di-GMP.

Cross-linking experiments were performed with 1 µM purified protein, an appropriate concentration of [^33^P]c-di-GMP (51, 67), and reaction buffer (50 mM NaH_2_PO_4_ [pH 6.5], 200 mM NaCl, and 1 mM dithiothreitol [DTT] or 20 mM Tris [pH 8.5], 200 mM NaCl, and mM DTT) as described in references 68 and 69 (see Text S1).

### Statistics.

For statistical comparisons, paired *t* tests were used if not stated otherwise. Calculations were performed with GraphPad Prism. Full experimental details are available in Text S1.
