# Supplementary figures and images for: Cohesive Properties of the Caulobacter crescentus Holdfast Adhesin Are Regulated by a Novel c-di-GMP Effector Protein
Source: mBio. 2017 Mar 21;8(2):e00294-17. doi: 10.1128/mBio.00294-17 (PMC5362036; doi:10.1128/mBio.00294-17)

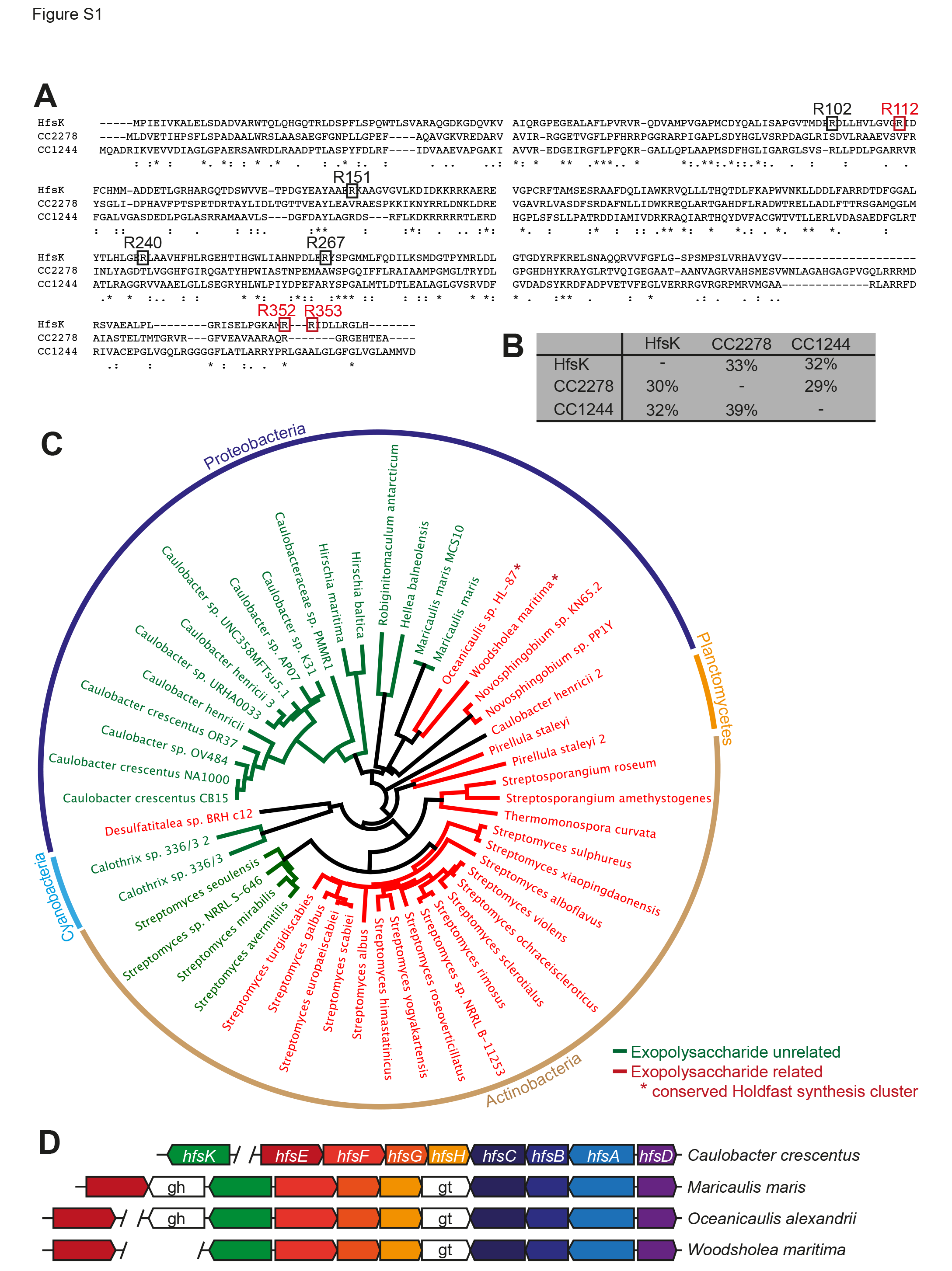

Supplement: FIG S1 [file mbo002173241sf1.tif]

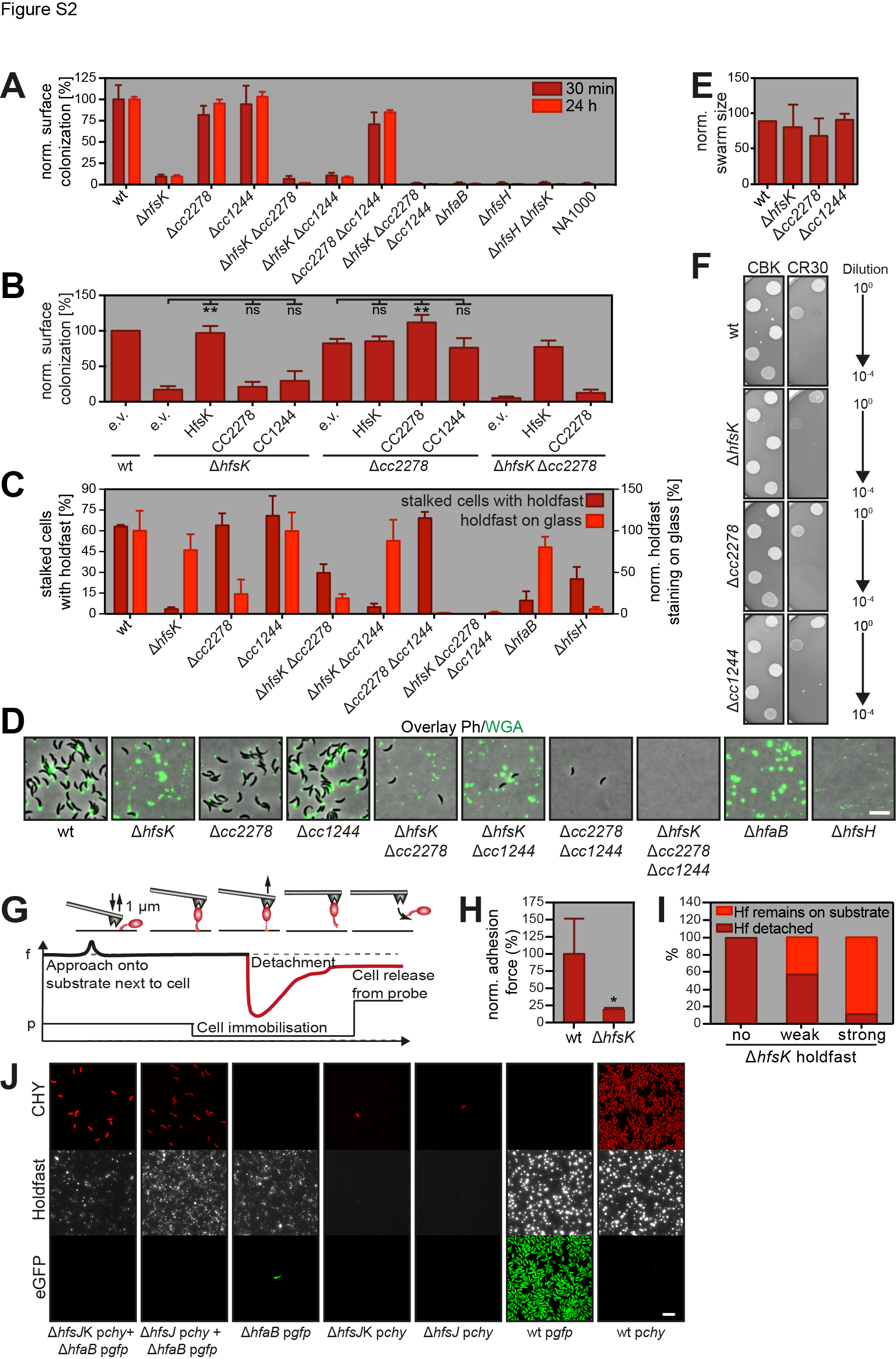

Supplement: FIG S2 [file mbo002173241sf2.tif]

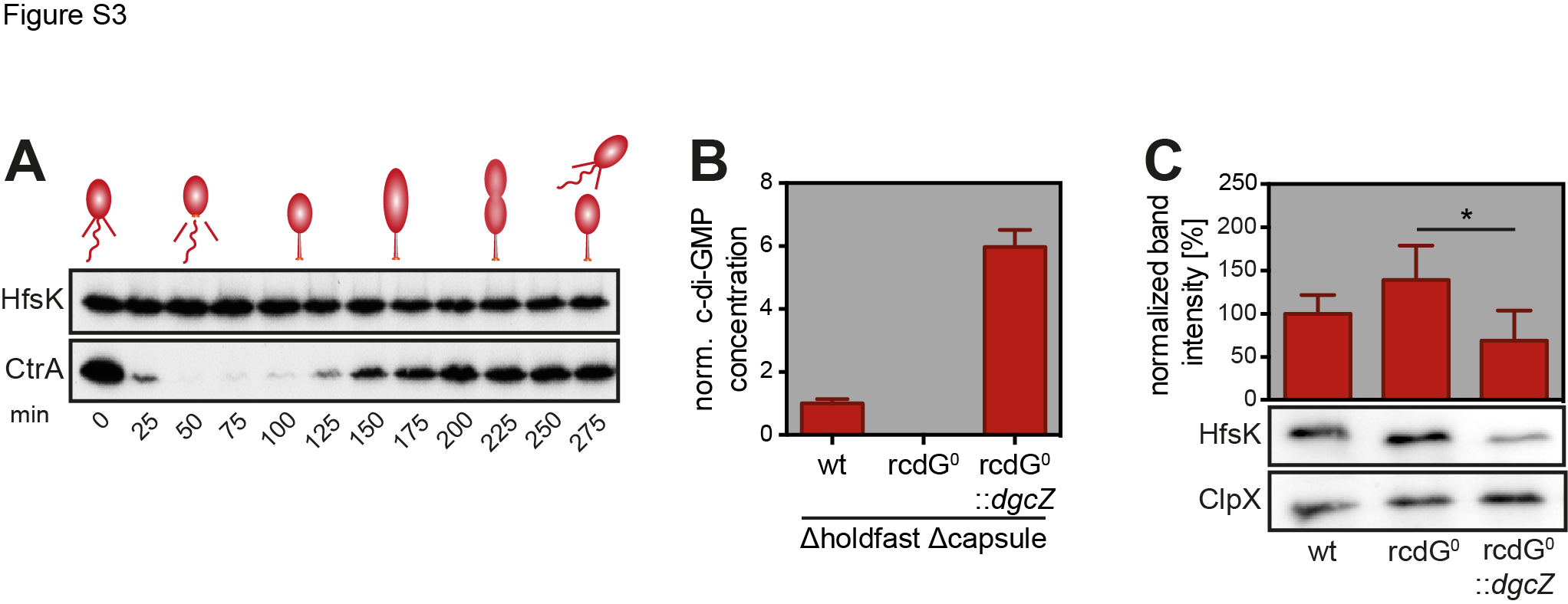

Supplement: FIG S3 [file mbo002173241sf3.tif]

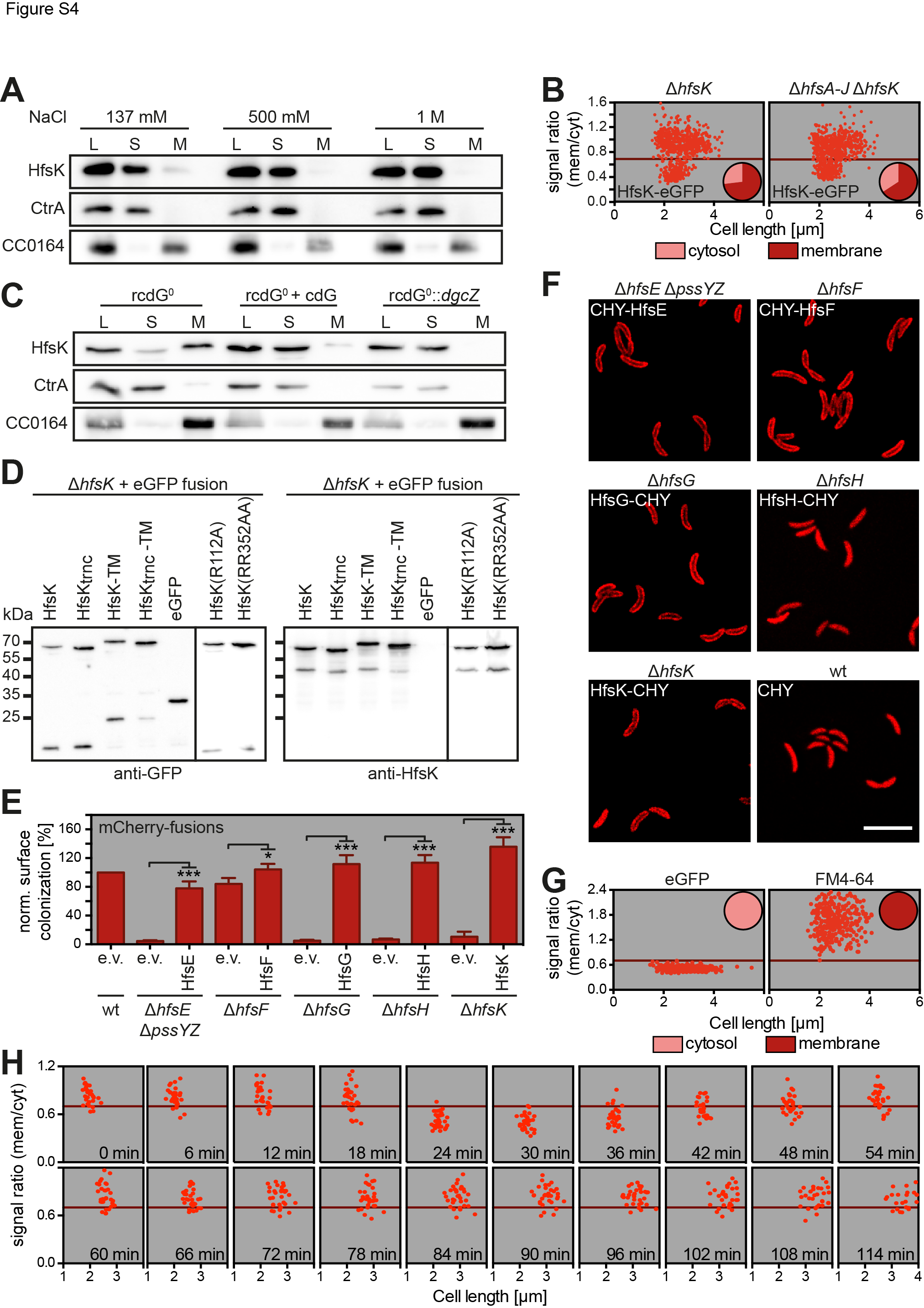

Supplement: FIG S4 [file mbo002173241sf4.tif]

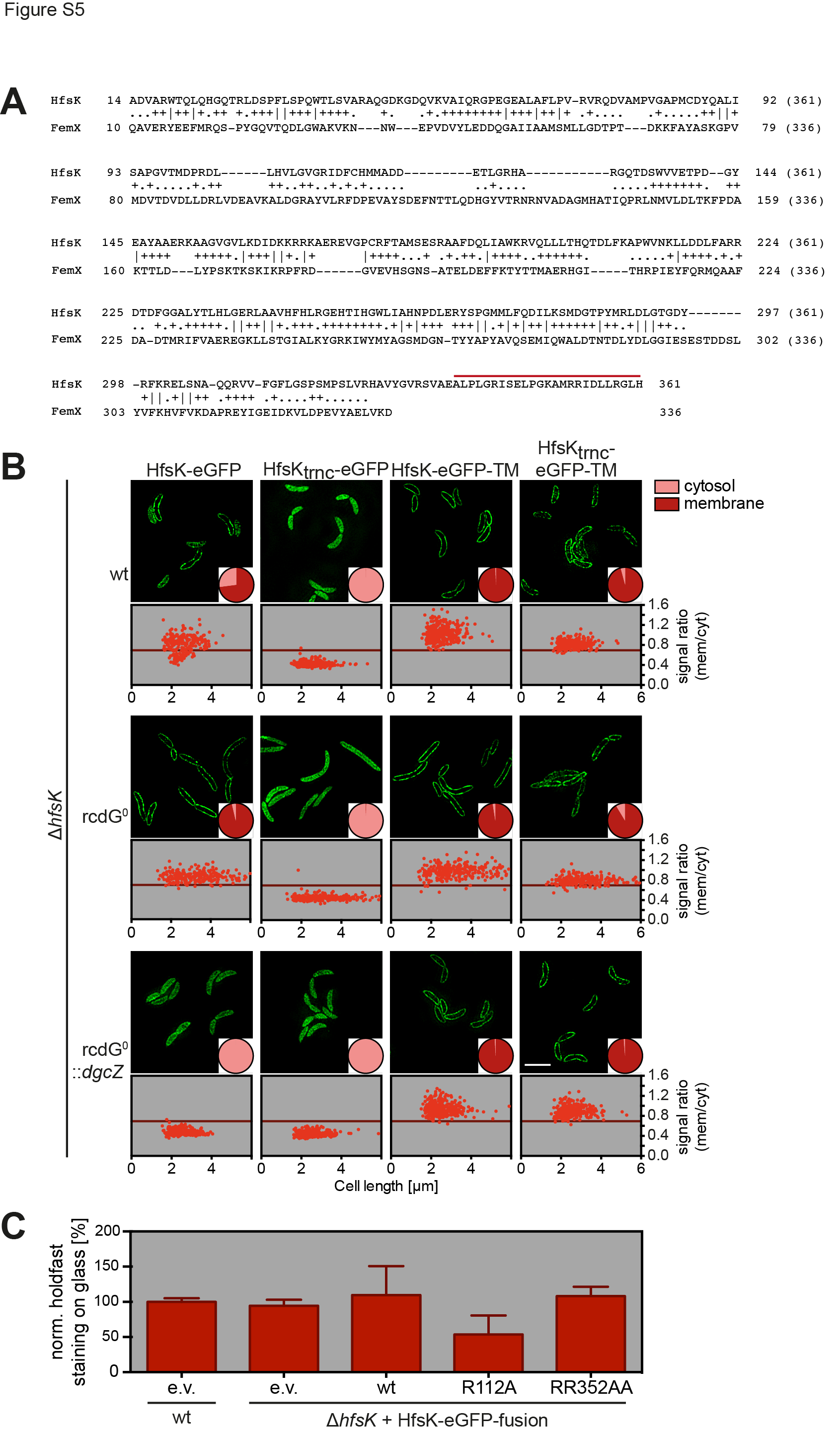

Supplement: FIG S5 [file mbo002173241sf5.tif]

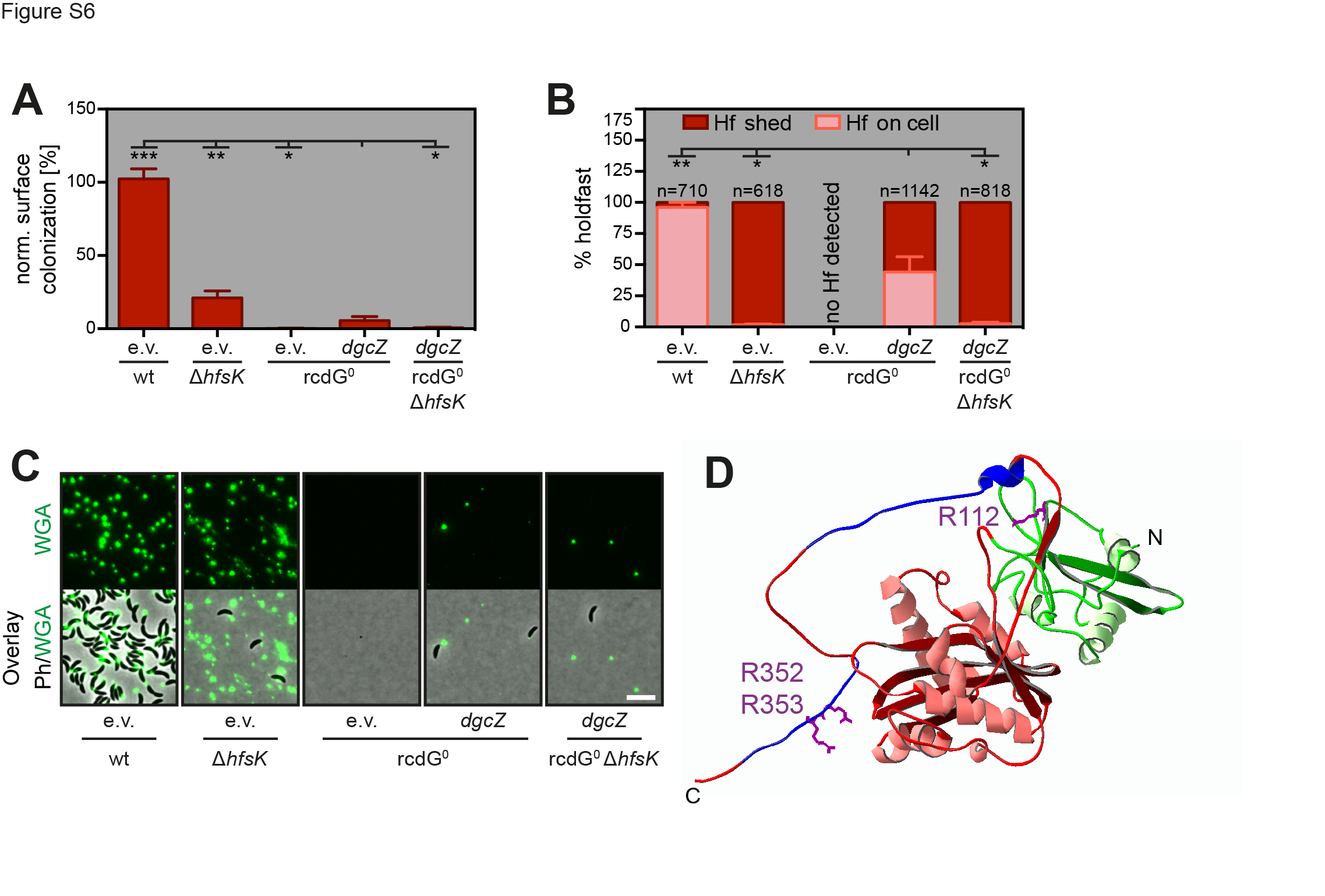

Supplement: FIG S6 [file mbo002173241sf6.tif]
