# Supplementary material for: Cohesive Properties of the Caulobacter crescentus Holdfast Adhesin Are Regulated by a Novel c-di-GMP Effector Protein
Source: mBio. 2017 Mar 21;8(2):e00294-17. doi: 10.1128/mBio.00294-17 (PMC5362036; doi:10.1128/mBio.00294-17)
Supplement: TEXT S1 [file mbo002173241s1.pdf]

Cohesive properties of the *Caulobacter crescentus* holdfast adhesin are regulated by a novel c-di-GMP effector protein

Kathrin S. Sprecher, Isabelle Hug, Jutta Nesper, Eva Potthoff, Mohamed-Ali Mahi, Matteo Sangermani, Volkhard Kaever, Torsten Schwede, Julia Vorholt, Urs Jenal

Material and Methods

Bacterial strains and growth conditions

All bacterial strains and plasmids used in this study are listed in Table S1 (below). *E. coli* strains were grown at 37°C or 30°C under aeration in Luria Broth (LB) medium supplemented with the appropriate antibiotic (solid/liquid in µg/ml: kanamycin 50/30; chloramphenicol 30/20) and the inducer Isopropyl β-D-1-thiogalacto-pyranoside (IPTG 0.3 or 0.75 mM) if required. *C. crescentus* strains were grown at 30°C under aeration in peptone yeast extract (PYE) or M2 minimal medium supplemented with 0.1% glucose (M2G). Media were supplemented with the appropriate antibiotic (solid/liquid in µg/ml: kanamycin 20/5; nalidixic acid 20/n.a.) or inducer (IPTG 0.2 or 0.5 mM, xylose 0.1%, vanillic acid 0.1 mM or 0.55 mM) if required. Media were solidified by addition of 1.5%, 0.75% or 0.3% agar for regular growth plates, top-agar and, motility plates, respectively. Optical density of cultures was measured at 600 nm (*E. coli*) or 660 nm (*C. crescentus*) with a photo spectrometer (Ultrospec 2100 pro, Amersham Biosciences, USA). If required cell cultures were synchronized using density gradient centrifugation as previously described (1).

Attachment assay

For 24-hour attachment assay 5 µl overnight culture were added to 155 µl appropriate medium in a 96 well polystyrene microtiter plate and grown for 24 h under aeration at 30°C. After absorbance was measured at 660 nm to confirm equal growth, the plates were rinsed thoroughly with water and, incubated for 30 min with 180 µl 0.1% (m/V) crystal violet/ 1% Methanol/ Isopropanol. The plates were rinsed again, dried, and the adherent crystal violet was dissolved in 200 µl 20% acetic acid for 15 min shaking at 30°C before absorption at 600 nm was measured.

For a 30-min attachment assay, overnight cultures were diluted to an OD_{660} of 0.06 in fresh medium, grown to an OD_{660} of 0.3-0.5. Cell numbers were adjusted to an OD_{660} of 0.3 before 160 µl culture was incubated into 96 well polystyrene microtiter plates and grown for 30 min under aeration at 30°C. Crystal violet staining was done as described above.

Fluorescence microscopy

Cells were harvested in mid-log phase (OD_{660} 0.3 - 0.5) and mounted on 1% agarose pads (Sigma, USA) in water for snap shots or in PYE containing appropriate supplements for time-lapse experiments. For 3D-SIM and time lapse microscopy samples were sealed with highly viscous silicone grease (Sigma Aldrich) to avoid agar shrinking.

Fluorescence, phase contrast (PH), and differential interference contrast (DIC) images were taken with a wide-field DeltaVision Core Olympus IX71 microscope (Applied Precision, USA) with SoftWoRx software and environmental chamber, equipped either with UPlanSApo 100x/ 1.40 oil

objective (Olympus, Japan) and a coolSNAP HQ-2 CCD camera (Photometrics, USA) or UPlan FL 100x/ 1.3 and UPlanSApo 100x/1.40 oil objectives (Olympus, Japan) and a pco.Edge sCMOS camera (PCO, Germany). Images with mCherry fusion proteins were taken with a Nikon Ti-E inverted motorized microscope with Perfect Focus System and PlanApo 100x/1.4 Oil Ph3 DM objective lens, SPECTRA × light engine (Lumencore), camera pco.Edge 4.2 (PCO, Germany), and VisiView software (Visitron Systems, Germany). Images showing protein localization were deconvolved using SoftWoRx and Huygens software.

3D-SIM imaging was performed on a microscope system (DeltaVision OMX-Blaze version 4; Applied Precision, USA) equipped with 405, 445, 488, 514, 568, and 642 nm solid-state lasers. Images were acquired using a PlanApoN 60x/1.42 oil objective lens (Olympus) and 4 liquid-cooled sCMOS cameras (pco.Edge, full frame 2560 x 2160; Photometrics). Exciting light was directed through a movable optical grating to generate a fine-striped interference pattern on the sample plane. The pattern was shifted laterally through five phases and three angular rotations of 60° for each z section. Optical z-sections were separated by 0.125 µm. The laser line 488 nm was used for 3D-SIM acquisitions. Multichannel imaging was achieved through sequential acquisition of wavelengths by separate cameras.

Raw 3D-SIM images were processed and reconstructed using the DeltaVision OMX SoftWoRx software package (Applied Precision (2, 3)). The resulting size of the reconstructed images was of 512 x 512 px from an initial set of 256 x 256 raw images. The channels were aligned in the image plane and around the optical axis using predetermined shifts as measured using a target lens and the SoftWoRx alignment tool. The channels were then carefully aligned using

alignment parameter from control measurements with 0.5 μm diameter multi-spectral fluorescent beads (Invitrogen, Molecular Probes).

Holdfast and membrane stain

To visualize membrane, mid log phase cultures were mounted on 1% agarose in PYE pads supplemented with 0.66 μg/ml FM4-64 dye (Molecular probes, USA). To visualize holdfast, mid log phase cultures were mixed with a final concentration of 1 μg/ml wheat germ agglutinin (WGA) coupled to Oregon green (Invitrogen, USA), incubated for 10 min, and visualized by fluorescence microscopy. For time-lapse microscopy experiments 2.66 μg/ml tetramethylrhodamine-WGA was added into the agarose pads.

Adherence to glass

To visualize adherent holdfast on glass a protocol was adapted from (4). 500μl/well overnight culture were diluted to an OD_{660} of 0.15 and incubated with round 12 mm borosilicate coverslips (Thermo Scientific, USA) in 24 well polystyrene plates for 2 h at 30°C under aeration. If plasmid induction was required, overnight grown cultures were diluted first 1:10 in medium containing the inducing agent and grown to mid-log phase, before cell numbers were adjusted to an OD_{660} of 0.15 and added to the coverslips. After incubation, the coverslip side facing upwards was stained for 15 min with 2.5 μg/ml WGA coupled to Oregon green, tetramethylrhodamine, or AlexaFluor®350, rinsed with water, and mounted on 1% agarose pads. Co-attachment experiments were performed on glass coverslips as described above, yet the co-cultured strains were mixed 1:1 to a final OD_{660} of 0.15 before incubation. For quantification 10

images were taken in random areas and the mean gray value was measured for each using the FIJI software (5). The mean gray value measured on glass slides prepared with the holdfast-minus NA1000 strains was subtracted to correct for background fluorescence.

Quantification of protein localization

Quantitative fluorescent signal measurements of individual cells were performed using a MATLAB based program developed in our group (WHISIT). WHISIT calculated average pixel fluorescent signal intensity for the membrane and cytoplasmic compartments. The membrane compartment was defined to enclose the first four intracellular pixel flanking the cell outline which was computed by Oufti (6) on phase contrast images, whilst the remaining intracellular pixels were defined as cytoplasmic compartment. The WHISIT program is available online at http://ch.mathworks.com/matlabcentral/fileexchange/61676-whisit).

Microfluidics

For the observation of bacteria under flow conditions they were grown in polydimethylsiloxane (PDMS) based microfluidics devices produced as previously described (7), consisting of a single channel of 10 mm length, 40 µm width, and 25 µm height connecting an in- and outlet. Mid-log phase cells were carefully filled into the channel before a constant flow (0.002 µl/s) of PYE medium supplemented with 1 µg/ml Oregon green-WGA was installed to allow growth. If necessary movies were corrected for bleaching using ImageJ Plugin CorrectBleach (V2.0.2, Kota Miura; Curtis Rueden; Mark Hiner; Johannes

Schindelin; Jens Rietdorf, Centre for Molecular and Cellular Imaging (CMCI), EMBL Heidelberg, [http://wiki.cmci.info/downloads/bleach_corrector])

Immunoblots

Cells were harvested and normalized in 1x SDS-SB (0.1 M Tris pH 6.8/ 5% Glycerol/ 0.2% sodium dodecyl sulfate (SDS)/ 1% β-Mercaptoethanol/ 0.025% Bromophenol blue) to the same OD_{660}. Samples were boiled for 5 min at 95°C, separated on 12% SDS-acrylamide gel electrophoreses (PAGE), and transferred onto PVDF-membranes (Immobilon-P, Millipore, USA). Proteins were detected using specific primary polyclonal antibodies (anti-CtrA 1:10,00, anti-CC0164 1:20,000, anti-ClpX 1:10,000, anti-GFP 1:800 (Invitrogen, USA)) and anti-rabbit secondary antibodies coupled to horseradish peroxidase (1:10,000, Dako, Denmark). After incubation with LumiGLO or LumiGLO reserve chemiluminescent substrate (KPL, USA) luminescence was detected using LAS-4000 luminescent image analyzer (Fujifilm, Japan). Immunoblot bands were quantified by measuring mean gray values with FIJI (5, 8).

StrepII-HfsK was purified as described below and injected into rabbits to raise polyclonal antibodies, (Josman LLC, USA). The serum was used in a 1:20,000 dilution.

Cell fractionation

150 ml mid log phase cultures were harvested by centrifugation (8000 xg, 20 min, 4°C) and washed in 50 ml PBS. The pellet was resuspended in 7 ml per gram wet weight CellFrac-Buffer (PBS/ 1x cOmplete mini EDTA-free Protease Inhibitor (Roche)/ 2.5 μg/ml DNAseI (Roche)), lysed using a French- pressure cell press at 1 bar (Thermo Electron corporation, USA), and spun to remove cell

debris (10 min, 18,000 xg, 4°C). To separate soluble from insoluble proteins, 1 ml of this cleared cell lysate was centrifuged at high speed (1 h, 100,000 xg, 4°C). The supernatant was removed and kept as soluble fraction whereas the pellet was washed in CellFrac-Buffer. The washed pellet was resuspended in 1 ml CellFrac-Buffer and kept as the pellet fraction. Cleared lysate, soluble and pellet fraction were diluted in 5x SDS-SB, boiled for 5 min at 95°C, and further analyzed using immunoblot.

Protein purification

pET28aStrepII plasmids expressing wild-type HfsK and mutant derivatives were transformed into *E. coli* Rosetta (DE3). Cells were grown at 30°C to an OD_{600} of 0.6, before the plasmid was induced with 0.75 mM IPTG for 4 h. Cells were harvested and resuspended in 50 mM NAH_2PO_4 / 300 mM NaCl supplemented with 1 µM Pepstatin / 1 mM DTT / 1x cOmplete mini (Roche) / 2.5 µg/ml DNAseI (Roche) and lysed using a French- pressure cell press at 1 bar. The StrepII-tagged proteins were purified from cleared lysates with Strep-Tactin Superflow plus resins (Qiagen) according to the manufacturer's protocol and used in the elution buffer or in 20 mM Tris pH 8.5/200 mM NaCl for further experiments.

The pET28aHis-HfsK plasmid was transformed into NiCo21(DE3) cells and grown at 37°C to an OD_{600} of 0.6. Protein expression was induced with 0.3 mM IPTG and incubation was continued at 22°C overnight. Cells were resuspended in 20 mM Tris pH 8.5/ 0.75 M NaCl/ 3 mM beta-mercaptoethanol/ 0.1%Tween−20 / 20 mM/ 1mM PMSF/ 1 µm pepstatin supplemented with protease inhibitor tablet (Roche) and disrupted using a microfluidizer (M-110L pneumatic, Microfluidics). Cleared lysate was applied to a 5 ml HisTrap HP column (GE

Healthcare) and the His-tagged protein was eluted with a gradient of elution buffer containing 20 mM Tris pH 8.5/ 0.5 M NaCl/ 3 mM beta-mercaptoethanol/ 0.1%Tween−20/ 500 mM imidazole. The eluted fractions containing HfsK protein were concentrated and injected on a Superdex 200 10/300 GL increase gel filtration column (GE Healthcare) equilibrated with 30 mM HEPES pH 7.4/ 0.3 M NaCl/ 3 mM beta-mercaptoethanol/ 5 mM MgCl2. The peak fractions corresponding to HfsK were collected and the concentration adjusted for ITC experiment.

Isothermal titration calorimetry

The interaction of HfsK with c-di-GMP was measured with a VP-ITC isothermal titration calorimeter from MicroCal with 13 μM HfsK in the cell and 211 μM c−di−GMP in the syringe (buffer: 30 mM HEPES pH 7.4, 0.3 M NaCl, 5 mM MgCl2, and 3 mM beta-mercaptoethanol). All solutions were thoroughly degassed and equilibrated to 22°C before filling into the calorimeter. The first injection of 3 μl was followed by 29 injections of 10 μl and the temperature of the calorimetric cell was maintained at 22°C. The delay between the injections was set to 6−7 min to ensure complete re-equilibration between subsequent injections. The observed data were analyzed using the MicroCal version of ORIGIN and fitted with the "One binding site model" of ORIGIN.

UV crosslinking with [^{33}P]c−di−GMP

[^{33}P]c−di−GMP was synthesized using [^{33}P]GTP (Hartmann Analytic, Germany) and the diguanylate cyclase DgcZ from *E. coli*. DgcZ purification and c-di-GMP

productions was performed as previously described (9, 10). Crosslinking experiments were performed as described in (11). In short, 1 µM purified protein, an appropriate concentration of [^{33}P] c−di−GMP, and reaction buffer (50 mM NaH2PO4 pH 6.5/ 200 mM NaCl/ 1 mM DTT or 20 mM Tris pH 8.5/200 mM NaCl/ 1 mM DTT) were mixed and incubated for 10 min at RT. In competition experiments the protein was pre-incubated with unlabeled competitor before [^{33}P]c-di-GMP was added. Samples were irradiated at 254 nm for 3 min at 4°C, mixed with 5x SDS-Sample Buffer, and boiled for 5 min. The samples were separated on 12% acrylamide gels using SDS-PAGE. The gels were dried and exposed to a phosphor screen that was scanned on a Typhoon FLA 7000 imaging system (GE Healthcare). Autoradiogram bands were quantified using FIJI (5, 8) by measuring the mean gray value and binding curves were fitted with GraphPad Prism 6.0.

SCFS measurements with FluidFM

In order to obtain clean substrates for the SCFS experiments, glass dishes (WillCo Wells B.V., The Netherlands) were sonicated in 2-propanol (Scharlau, Spain) and subsequently in ultra-pure water for 10 min at room temperature in a Branson 2210 Ultrasound bath and dried under nitrogen gas stream right before use. Cultures grown overnight in PYE were diluted to an OD_{600} of 0.001 and together with a final concentration of 0.1 µg/ml Oregon green-WGA were added to the clean glass dishes. SCFS measurements were started right away and could last several hours. Thus, measurements comprise this range of surface contact times for all strains.

Preparation and calibration of the cantilever was performed as described earlier (12). Briefly, rectangular, hollow silicon nitride cantilevers containing a hollow pyramid at the free-end (Cytosurge AG, Switzerland) were 36 μm wide, 150 μm long, and had a channel height of 1 μm, resulting in a stiffness of about 2.5 N/m. Circular 300 – 700 nm diameter openings at the pyramidal apex were drilled by focused ion beam after sloping the pyramid's tip to compensate for the 10° tilt angle of the AFM probe holder (13). Prior to the experiments the probes were plasma-cleaned for 30 s (Plasma Cleaner PDG-32G, Harrick Plasma, USA) and covered with an antifouling coating of 0.5 mg/ml poly-L-lysine (PLL) (20 kDa) that was grafted with poly-ethylene glycol (PEG) (2 kDa) (PLL-*g*-PEG) (Surface Solution SuSoS AG, Switzerland) in filtered ultra-pure water (14). FluidFM probes were coated from the in- and outside with PLL-*g*- PEG for 1 h and subsequently washed in filtered PBS for 5 min (15). Cantilever sensitivity was calibrated using software-implemented scripts based on the formalism described by Sader and colleagues (16).

A FluidFM connected via tubing to a pressure controller (Cytosurge AG, Zürich and Nanosurf AG, Liestal, Switzerland) was mounted on an Axio Observer D1 inverted microscope (Carl Zeiss, Jena, Germany).

For previously developed FluidFM applications, the probe was directly approached onto adherent cells prior to underpressure based cell immobilization to the cantilever and subsequent cell detachment of the substrate (12, 14). However, a direct approach onto the cell is not possible for *C. crescentus*, as the flexible parts of the cells are pushed away during the downward movement of the probe. In order to achieve a defined distance above the substrate, the pyramidal tip was approached next to a *Caulobacter* cell and

retracted until the desired separation was achieved. Subsequently, to immobilize the cell to the cantilever, the probe was moved in x-y directions above the target cell and 0.8 atm underpressure was applied for reversible cell immobilization at the pyramidal tip opening. Subsequently the probe, together with the attached cell was retracted at a piezo velocity of 1 μm/s, while forces were recorded. Underpressure was maintained during this process until the bacterium was completely detached from the substrate. Subsequently the bacterium was released from the cantilever by an overpressure pulse of 1 atm. SCFS data were analyzed with SPIP software (Image Metrology A/S, Hørsholm, Denmark).

Statistics

For statistical comparison, paired t-tests were used if not stated otherwise. Calculations were performed with GraphPad Prism.

C-di-GMP quantification

C-di-GMP was extracted from 20 ml liquid culture of strains harboring a deletion in *hfsA* (holdfast^{-}) and *cc00471* (capsule^{-}) to prevent EPS based clogging of the HPLC column. Extraction and quantification was performed as previously described (17, 18).

Alignments, phylogenetic analysis and structural model

Sequence homologs of HfsK were obtained using Blast search (19) (database accessed July 2015) and the phylogenetic tree was computed with Geneious 7.1.7 using global alignment with free end gaps and neighbor joining methodology.

The structural model of HfsK was created with the MPI bioinformatics Toolkit (20) using structural homology search with HHpred (21), followed by structure prediction with the build-in modeller function (22). Structure based alignment was adapted from the HHpred structural homology search output and multiple sequence alignments were created using MUSCLE (23).

Phage and motility assay

Phage lysates were prepared based on (24). In short, mid-log phase bacterial culture was mixed with phage lysate, incubated for 10 min at room temperature, mixed with prewarmed PYE top-agar (0.75%) and poured onto a PYE plate. After overnight growth at 30 °C, 5 ml CPB buffer (10 mM Tris pH 7.5 / 1 mM $MgSO_4$ / 1 mM $CaCl_2$) were added and incubated again overnight at 4 °C. The next day, lysed cells were scraped from the plate, mixed with 150 µl chloroform and incubated for 1 h. After centrifugation (5,000 xg, 10 min), another 150 µl chloroform were added to the cleared supernatant to get the phage lysate stock.

To assess phage susceptibility, 200 µl stationary phase culture was mixed with 2.5 ml PYE top-agar (45°C) and poured on top of PYE plates. Onto the solidified agar 5 µl of serial dilutions of phage lysate were spotted. Phage lysates were made in CPB buffer. The plates were incubated for 1 day at 30°C and scanned (ScanMaker i800, Microtek International). To score motility, semi-solid PYE 0.3% agar plates were inoculated with a single colony and incubated for 3 days in a humidified chamber at 30°C. The plates were scanned and colony size was measured using FIJI (5, 8).

Construction of plasmids

For pNPTS138-based deletion plasmids roughly 500 bp up-and downstream of the target gene were amplified from genomic CB15 DNA with PCR, cut with restriction enzymes as indicated in Table S1 (below), and ligated at the same time into EcoRI/ HindIII (or in case of pNPTS-Δ*2278*: SpeI/ EcoRI) cut pNPTS138 vector. In case of pNPTS138-Δ*hfsK* and the inner primers (3585/35876) have extensions that are complementary to each other, thus the two fragments were fused using SOE-PCR and primers 8584/3587. The fused products were cut with EcoRI/HindIII and ligated in the likewise cut pNPTS138 vector.

StrepII-fusions were generated using the subcloning vector pET28A-StrepII-MCS which was generated by annealing of two complementary oligonucleotides encoding the *strepII* tag (primer 3287/3288), followed by restriction enzyme digest with NcoI/ BamHI and ligation into the likewise cut pET28a vector. pKaS105 was generated by amplification of *hfsK*$_{\mathrm{R352A,R353A}}$ from pKaS95 with PCR followed by restriction enzyme digest using primers and enzymes as indicated in Table S1 below and ligation into the likewise cut pET28A-StrepII-MCS subcloning vector. pET28strepII-hfsK and pKaS93 were generated by amplification of *hfsK* and *hfsK*$_{\mathrm{trnc}}$ from genomic CB15 DNA with PCR followed by restriction enzyme digest using primers and enzymes as indicated in Table S1 below and ligation into the likewise cut pET28A-StrepII-MCS subcloning vector. pKaS84 was generated similarly except that in addition SOE-PCR was used to introduce mutation R112A with the mutagenic primers 8890/8891.

pKaS2, pKaS1, pKaS9, pKaS22, pKaS67, pKaS95, pKaS106, pKaS111, pKaS12, pMT687-*hfsK*, pKaS113, and pKaS114 were generated by amplification of the

gene of interest from genomic CB15 DNA with PCR followed by restriction enzyme digest using primers and enzymes as indicated in Table S1 below and ligation into the likewise cut pMT552, pMT590, or pMT697 vector. pKaS77 was generated by using primers 8828/8830 as well as template pMT552 to amplify *egfp* and primers 8829/8831 as well as the template pNPTS138-*hfsK-tm* (see below) to amplify *secE-tm* with a linker region. As Primer 8829 and 8830 have extensions that are complementary to each other, the two fragments were fused using SOE-PCR generating *egfp-tm* with primers 8828/8831. This was cut with EcoRI and NheI and ligated into the cut pKaS22 vector. pNPTS138-*hfsK-tm* was generated by amplifying with PCR from genomic DNA the last 500 bp of *hfsK* with primer 4033/8396, *tm* region of *secE* with primer 8395/8398 and 500 bp downstream region of *hfsK* with primer 8397/4036. As the primer pairs 8395/ 8396 and 8397/8398 have extensions that are complementary to each other, SOE-PCR was used to first fuse the *hfsK* fragment with *secE-tm* using primers 4033/8398 and then the resulting *hfsK-tm* fragment with the downstream fragment of *hfsK* using primers 4033/4036. The resulting product was cut with HindIII and EcoRI and ligated into the likewise cut pNPTS138 vector.

pKaS90 was generated by extracting *hfsK*$_{\text{trnc}}$ from pKaS67 by restriction enzyme digest with NdeI/ KpnI followed by ligation into the linearized vector pKaS77.

Table S1: Strains and plasmids used in this study

Plasmids

Plasmid	Description	Source/ Reference
pET28a	Kan^R, pBR322 based high copy vector with promoter from T7 bacteriophage	Novagen®
pNPTS138	Kan^R, suicide vector with *sacB* and *oriT*, for generation of genomic mutations by allelic exchange	D. Alley

Plasmid	Description	Source/ Reference
pMT552	Kan^R, pVGFPC-2, *vanA*-P$_{van}$-MCS-*egfp*; for generating C-terminal egfp fusions inserted in the *vanA* locus expressed from P$_{van}$ or for expression of *egfp* alone	(25)
pMT585	Kan^R, pXGFPC-2, *xylX*-P$_{xyl}$-MCS-*egfp*; for generating C-terminal egfp fusions inserted in the *xylX* locus expressed from P$_{xyl}$ or for expression of *egfp* alone	(25)
pMT590	Kan^R, pXCHYC-2, *xylX*-P$_{xyl}$-MCS-*chy*; for generating C-terminal *mcherry* fusions inserted in the *xylX* locus expressed from P$_{xyl}$ or for expression of *mcherry* alone	(25)
pMT697	Kan^R, pXCHYN-2, *xylX*-P$_{xyl}$-*chy*-MCS; for generating N-terminal *mcherry* fusions inserted in the *xylX* locus expressed from P$_{xyl}$ or for expression of *mcherry* alone	(25)
pMT687	Kan^R, RK2 based low-copy vector with P$_{xyl}$	(25)
pSRK	Kan^R, pBBR1MCS-2 based high copy vector with P$_{lac}$	(26)
pET28strepII-*hfsK*	pET28a plasmid encoding *strepII-hfsK*	this study
pKaS93	pET28a plasmid encoding *strepII-hfsK*$_{trnc}$	this study
pKaS84	pET28a plasmid encoding *strepII-hfsK*$_{\mathrm{R112A}}$	this study
pKaS105	pET28a plasmid encoding *strepII-hfsK*$_{\mathrm{R352A,\ R353A}}$	this study
pET28his-*hfsK*	pET28a plasmid encoding 6x*his-hfsK*	M. Meier
pNPTS138-Δ*hfsK*	pNPTS138 derivative for generation of Δ*hfsK* mutation	Y. Cohen
pNPTS138-Δ*2278*	pNPTS138 derivative for generation of Δ*cc2278* mutation	Y. Cohen
pKaS110	pNPTS138 derivative for generation of Δ*1244* mutation	this study
pSA223	pNPTS138 derivative to integrate a P$_{lac}$ driven *dgcZ-3xflag* into the intergenic region of *cc3065* and *cc3066*	(27)
pSA81	pNPTS138 derivative for generation of Δ*pdeA* mutation	(28)
pSA156	pNPTS138 derivative for generation of Δ*cc0091* mutation	(27)
pNPTS138-Δ*00471*	pNPTS138 derivative for generation of Δ*ccna00471* mutation	this study
pSA79	pNPTS138 derivative for generation of Δ*cc1086* mutation	(27)
pSA90	pNPTS138 derivative for generation of Δ*cc3148* mutation	(27)
pNPTS138Δ*hfaA*	pNPTS138 derivative for generation of Δ*hfaA* mutation	(29)
pNPTS138-Δ*hfaB*	pNPTS138 derivative for generation of Δ*hfaB* mutation	this study
pNPTS138Δ*hfaD*	pNPTS138 derivative for generation of Δ*hfaD* mutation	(29)
pKaS59	pNPTS138 derivative for generation of Δ*hfsA* mutation	this study
pKaS25	pNPTS138 derivative for generation of Δ*hfsE* mutation	this study
pKaS26	pNPTS138 derivative for generation of Δ*hfsF* mutation	this study
pNPTS138Δ*hfsG*	pNPTS138 derivative for generation of Δ*hfsG* mutation	(30)
pNPTS138Δ*hfsH*	pNPTS138 derivative for generation of Δ*hfsH* mutation	(30)
pNPTS138Δ*hfsI*	pNPTS138 derivative for generation of Δ*hfsI* mutation	(30)
pDM25	pNPTS138 derivative for generation of Δ*hfsJ* mutation	D. Meyer
pKaS52	pNPTS138 derivative for generation of Δ*hfsABCDEFGH* mutation	this study
pNPTS138Δpss*Y*	pNPTS138 derivative for generation of Δ*pssY* mutation	(30)
pNPTS138Δ*pssZ*	pNPTS138 derivative for generation of Δ*pssZ* mutation	(30)
pKaS22	pVGFPC-2 derivative for expression of *hfsK-egfp* from P$_{van}$	this study
pKaS67	pVGFPC-2 derivative for expression of *hfsK*$_{trnc}$-*egfp* from P$_{van}$	this study
pKaS77	pVGFPC-2 derivative for expression of *hfsK-egfp-tm* from P$_{van}$	this study
pKaS90	pVGFPC-2 derivative for expression of *hfsK*$_{trnc}$-*egfp-tm* from P$_{van}$	this study
pKaS95	pVGFPC-2 derivative for expression of *hfsK*$_{\mathrm{R352A,R353A}}$-*egfp* from	this study

Plasmid	Description	Source/ Reference
	P_{van}	
pKaS106	pVGFPC-2 derivative for expression of *hfsK*$_{R112A}$-*egfp* from P_{van}	this study
pKaS111	pXCHYC-2 derivative for expression of *mcherry-hfsE* from P_{xyl}	this study
pKaS112	pXCHYC-2 derivative for expression of *mcherry-hfsF* from P_{xyl}	this study
pKaS2	pXCHYN-2 derivative for expression of *hfsG-mcherry* from P_{xyl}	this study
pKaS1	pXCHYN-2 derivative for expression of *hfsH-mcherry* from P_{xyl}	this study
pKaS9	pXCHYN-2 derivative for expression *of hfsK-mcherry* from P_{xyl}	this study
pMT687-hfsK	pMT687 derivative for expression of *hfsK* from P_{xyl}	this study
pKaS113	pMT687 derivative for expression of *cc2278* from P_{xyl}	this study
pKaS114	pMT687 derivative for expression of *cc1244* from P_{xyl}	this study
pTB4	pSRK derivative for expression of *dgcZ* from P_{lac}	(27)

Strains

Strain	Genotype	Description	Source/ Reference
E. coli			
DH5alpha	F^- *endA1* hsdR17 ($r_K^- m_K^+$) *glnV44 thi-1 recA1 gyrA96 relA1* Δ(*lacZYA-argF*)U169 *deoR* Φ80*dlacZ*ΔM15)	High efficiency transformation strain	(31)
DH10B	F^- *mcrA* Δ(mrr-hsdRMS-mcrBC) Φ80d*lacZ*ΔM15 *lacX74 endA1 recA1* Δ(*ara, leu*)7697 *araD139 galU galK nupG λ*-	Used in conjugations transferring plasmids to *C. crescentus* with help of MT607 as plasmid donor	Invitroge n
S17-1	RP4-2, Tc::Mu, KM-Tn7	Used in conjugations transferring plasmids to *C. crescentus* as plasmid donor	(32)
MT607	pRK600 (cam^R)	Conjugation helper strain with F-plasmid	
Rosetta (DE3)	F^- *ompT hsdS*$_B$(r_B^- m_B^-) *gal dcm* (DE3) pLysSRARE2 (Cam^R)	BL21 derivative; compatible with T7 expression vectors	Novagen®
NiCo21 (DE3) pLys	*can::CBD fhuA2 [lon] ompT gal* (*λ DE3*) *[dcm] arnA::CBD slyD::CBD glmS6Ala ΔhsdS λ DE3 = λ sBamHIo ΔEcoRI-B int::*(*lacI::PlacUV5::T7 gene1*) *i21 Δnin5*	BL21 derivative; compatible with T7 expression vector; minimizes *E. coli* protein contamination in immobilized metal affinity chromatography	New England Biolabs
C. crescentus			
NA1000	CB15N	*C. crescentus* laboratory strain derived from CB15	(33)
CB15	CB15	*C. crescentus* wild-type ATCC19089	(34)
UJ5990	Δ*hfsK*	Markerless deletion of *hfsK* in CB15 using pNPTS138-Δ*hfsK*	this study
UJ6136	Δ*cc2278*	Markerless deletion of *cc2278* in CB15 using pNPTS138-Δ*2278*	Y. Cohen
UJ6237	Δ*cc1244*	Markerless deletion of *cc1244* in CB15 using pKaS110	this study
UJ6155	ΔhfsK Δ*cc2278*	Markerless deletion of *cc2278* in CB15 Δ*hfsK* using pNPTS138-	this study

Strain	Genotype	Description	Source/ Reference
		Δ*2278*	
UJ6238	ΔhfsK Δ*cc1244*	Markerless deletion of *cc1244* in CB15 Δ*hfsK* using pKaS110	this study
UJ6239	Δcc2278 Δ*cc1244*	Markerless deletion of *cc1244* in CB15 Δ*cc2278* using pKaS110	this study
UJ6241	Δ*hfsK* Δ*cc2278* Δ*1244*	Markerless deletion of *cc1244* in CB15 Δ*hfsK* Δ*2278* using pKaS110	this study
UJ7113	Δ*hfsK* Δ*hfsH*	Markerless deletion of *hfsH* in CB15 Δ*hfsK* using pNPTS138Δ*hfsH*	this study
UJ9545	Δ*hfaA*	Markerless deletion of *hfaA* in CB15 using pNPTS138Δ*hfaA*	this study
UJ6951	Δ*hfaB*	Markerless deletion of *hfaB* in CB15 using pNPTS138-ΔhfaB	this study
UJ9547	Δ*hfaD*	Markerless deletion of *hfaD* in CB15 using pNPTS138Δ*hfaD*	this study
UJ9583	Δ*hfaA* Δ*hfaD*	Markerless deletion of *hfaA* in CB15 Δ*hfaD* using pNPTS138Δ*hfaA*	this study
UJ7112	Δ*hfsH*	Markerless deletion of *hfaH* in CB15 using pNPTS138Δ*hfsH*	this study
UJ8820	Δ*hfsE* Δ*pssY* Δ*pssZ*	Markerless deletion of *hfsE*, *pssY* and *pssZ* in CB15 using pKaS25, pNPTS138Δ*pssY* and pNPTS138Δ*pssZ*	this study
UJ8162	Δ*hfsF*	Markerless deletion of *hfsF* in CB15 using pKaS26	this study
UJ7847	Δ*hfsG*	Markerless deletion of *hfsG* in CB15 using pNPTS138Δ*hfsG*	this study
UJ9470	Δ*hfsABCDEFGH* Δ*hfsI* Δ*hfsJ* Δ*hfsK*	Markerless deletion of *hfsABCDEFGH* operons, *hfsI*, *hfsJ*, and, *hfsK* in CB15 using pKaS52, pNPTS138Δ*hfsI*, pDM25 and, pNPTS138-Δ*hfsK*	this study
UJ5100	cdG^0; Δ*cc0655* Δ*cc0740* Δ*cc0857* Δ*cc0896* Δ*dgcB* Δ*pleD* Δ*cc3094* Δ*dgcA*	Markerless deletion of *cc0655 cc0740 cc0857 cc0896 dgcB pleD cc3094 dgcA* in CB15	(27)
UJ8732	rcdG^0; (Δ*cc0091* Δ*cc0655* Δ*cc0740* Δ*cc0857* Δ*cc0896* Δ*cc1086* Δ*dgcB* Δ*pleD* Δ*cc3094* Δ*cc3148* Δ*dgcA* Δ*pdeA*)	Markerless deletion of *cc0091*, *cc1086*, *cc3148*, *pdeA* in CB15 cdG^0 using pSA156, pSA79, pSA81, and pSA90	this study
UJ8781	rcdG^0+*dgcZ*; (Δ*cc0091* Δ*cc0655* Δ*cc0740* Δ*cc0857* Δ*cc0896* Δ*cc1086* Δ*dgcB* Δ*pleD* Δ*cc3094* Δ*cc3148* Δ*dgcA* Δ*pdeA* P_{lac}-*ydeH-3xflag*)	Chromosomal integration of P_{lac} driven *dgcZ* into CB15 rcdG^0 using pSA223	this study
UJ8877	rcdG^0 Δ*hfsK*	Markerless deletion of *hfsK* in CB15 rcdG^0 using pNPTS138-ΔhfsK	this study
UJ8878	rcdG^0+*dgcZ* Δ*hfsK*	Markerless deletion of *hfsK* in CB15 rcdG^0+*dgcZ* using pNPTS138-Δ*hfsK*	this study
UJ4463	Δ*pleD*	Markerless deletion of *pleD* in CB15	(28)
UJ9078	Δ*pleD* Δ*hfsK*	Markerless deletion of *hfsK* in Δ*pleD* using pNPTS138-ΔhfsK	this study
UJ9633	Δ*hfsA* Δ*ccna00471*	Markerless deletion of *hfsA* and	this study

Strain	Genotype	Description	Source/ Reference
		ccna0471 in CB15 using pKaS59 and pNPTS138-Δ*00471*	
UJ9634	rcdG^{0} Δ*hfsA* Δ*ccna00471*	Markerless deletion of *hfsA* and *ccna0471* in CB15 rcdG^{0} using pKaS59 and pNPTS138-Δ*00471*	this study
UJ9635	rcdG^{0}+*dgcZ* Δ*hfsA* Δ*ccna00471*	Markerless deletion of *hfsA* and *ccna00471* in CB15 rcdG^{0}+*dgcZ* using pKaS59 and pNPTS138-Δ*00471*	this study
UJ7870	Δ*hfsJ*	Markerless deletion of *hfsJ* in CB15 using pDM25	this study
UJ7871	Δ*hfsJ* Δ*hfsK*	Markerless deletion of *hfsJ* in CB15 Δ*hfsK* using pDM25	this study
**Phages**			
ΦCbK		Bacteriophage that uses pili of *C. crescentus* for infection	(35)
ΦCR30		Bacteriophage that uses the paracrystalline surface- layer proteins of *C. crescentus* as a receptor	(36)

Primers

Primer	Sequence	Restriction site	Used for plasmid
1380	GAATTCTTCGACCGTTCCCAGCCC	EcoRI	pDM25
1381	GGATCCCGCTGTCCAGACGCTCTA	BamHI	pDM25
1382	GGATCCTGAGGAACGAACATCTCCGCAG	BamHI	pDM25
1383	AAGCTTCGACAAGGACGGCCAGAAGGA	HindIII	pDM25
3287	ATATACCATGGGATGGAGCCACCCGCAGTTCGAAAAAGGATCCAAGCTT	n/a	pET28strepII-*hfsK*/ pKaS93/ pKaS84/ pKaS105
3288	AAGCTTGGATCCTTTTTCGAACTGCGGGTGGCTCCATCCCATGGTATAT	n/a	pET28strepII-*hfsK*/ pKaS93/ pKaS84/ pKaS105
3300	AAGGATCCCCCATCGAAATCGTCAAAGC	BamHI	pET28strepII-*hfsK*/ pKaS93/ pKaS84/ pKaS105
3301	AAGCAAGCTTTCAGTGCAGTCCGCGCAGCA	HindIII	pET28strepII-*hfsK*/ pKaS84/ pKaS105
3584	AGAGAAGCTTGCAAGATCACCTCGCCGCGT	HindIII	pNPTS-Δ*hfsK*
3585	TTGCCCATCGAAATCGTCAAACTGCTGCGCGGACTGCACTGA	n/a	pNPTS-Δ*hfsK*
3586	TCAGTGCAGTCCGCGCAGCAGTTTGACGATTTCGATGGGCAA	n/a	pNPTS-Δ*hfsK*
3587	CTCTGAATTCCGCTGTTCGAGCGCATGGCC	EcoRI	pNPTS-Δ*hfsK*
3942	AGACGACCATATGCCCATCGAAATCGTCAAAGC	NdeI	pKaS22/ pKaS67/ pKaS95/ pKaS106/ pKaS9
3943	GTGGTACCTCAGTGCAGTCCGCGCAGCA	KpnI	pMT687-hfsK
3984	ATATACTAGTATCGTGGTGATAGAGGCTCAC	SpeI	pNPTS-Δ*2278*

3985	ATATAAGCTTCTGCAATCGACAGGCCATTCC	HindIII	pNPTS-Δ*2278*
3986	ATATAAGCTTGCCTGATGGCGCGCGTCACGG	HindIII	pNPTS-Δ*2278*
3987	ATATGAATTCCGGCGACGAGACCGAAGACTG	EcoRI	pNPTS-Δ*2278*
4033	GACAAAGCTTTGCTGACCCACCAGACCGAC	HindIII	pKaS77
4036	TAGAATTCTTCGAGCGCATGGCCGAGGC	EcoRI	pKaS77
4088	GTCCAAGCTTCGACCCCCTGATCGACTG	HindIII	pKaS110
4089	ATGGTACCCGCATTAGGCCTTAAGCATC	KpnI	pKaS110
4090	GAGGTACCGGTCTGGCGATGATGGTG	KpnI	pKaS110
4091	TGAATTCCCTGAGCGATTTCCAGCTT	EcoRI	pKaS110
4164	CTTAAGGTCATATGCAGCCGGCGATCCATCTTT	NdeI	pKaS113
4165	ACTGGTACCATCAGGCCTCCGTGTGCTCT	KpnI	pKaS113
4587	AGACGACCATATGCAGGCCGACCGTATCAAGGT	NdeI	pKaS114
4596	TCAGGTACCTGAGGAGAGGCTACCGAGG	KpnI	pKaS114
4726	TAGAATTCGCCTGGAAAAGAAGACCAAG	EcoRI	pNPTS-Δ*00471*
4727	ATGGTACCGATCACGTCATTCGGCATTG	KpnI	pNPTS-Δ*00471*
4728	CAGGTACCCACTTCCTCTCGGCCCAATA	KpnI	pNPTS-Δ*00471*
4729	CATTGAAGCTTGACTGCGAACGATCGCTAGA	HindIII	pNPTS-Δ*00471*
5181	GGAATTCGACTTCTATCTAGGGGCTCG	EcoRI	pNPTS138-Δ*hfaB*
5182	GAGGTACCTGTGCGCTTGACCATCATTT	KpnI	pNPTS138-Δ*hfaB*
5183	GAGGTACCGATATCCGTGATGCTAAGCG	KpnI	pNPTS138-Δ*hfaB*
5184	GCTAGAAGCTTTCACGTTGATGTTGTTGCCC	HindIII	pNPTS138-Δ*hfaB*
5592	GTGGTACCAGCAGTACTTCCGCGACCT	KpnI	pKaS52
5593	GGAATTCCAGAGTCCTGTTCGGTCAGC	EcoRI	pKaS52
5684	GAGATTACCATATGAACGCGCCCGTCAACGA	NdeI	pKaS2
5685	CCGGTACCGACGGCCTCGCTGTAGAGCG	KpnI	pKaS2
5686	GAGATTATCATATGCCGATGGAATTCGAGAA	NdeI	pKaS1
5687	CCGGTACCGAGCCCGATCCGCCGCG	KpnI	pKaS1
5692	TAGGTACCGTTTTCCCAACGACGAGCAT	KpnI	pKaS111
5693	TAGAATTCCTAGCGCACGGCGGACCGAT	EcoRI	pKaS111
5694	TAGGTACCTTCTGGCGCGGCGTCCTCGG	KpnI	pKaS112
5695	TAGAATTCTCATGCGGCTTGCGCCTTTC	EcoRI	pKaS112
6119	AGCAGGTACCGTGCAGTCCGCGCAGCAGGT	KpnI	pKaS22/ pKaS106/ pKaS9
6874	TTAACGGTACCCGCCGGATCCAGCACGCGCG	KpnI	pKaS26
6875	TTAACAAGCTTCTTCAACAACGAGGCGATTC	HindIII	pKaS26
6876	CGTTATAGAATTCAAAAGCCCTCGTCGAAGC	EcoRI	pKaS26
6877	TTAGTGGTACCCGTCGAAAGGCGCAAGCCGC	KpnI	pKaS26
6878	TTAACGGTACCGCTGGCCCCGATCCCCATCA	KpnI	pKaS25/ pKaS52
6879	TTAACAAGCTTCGAACCCTCGATACCCTTT	HindIII	pKaS25/ pKaS52
6880	TTAAAGGAATTCGCCGGTCATGAACTTCAACT	EcoRI	pKaS25
6881	TTAAAGGTACCGGGGATCGGTCCGCCGTGCG	KpnI	pKaS25
8373	TGAGGTACCGGTGAGCGCTTTGGTCGCCT	KpnI	pKaS59
8374	ATTGAAGCTTGACGGTCTGGTCCATGTGC	HindIII	pKaS59
8375	TGTAAGAATTCCCTTCACCGAAATCTGCAC	EcoRI	pKaS59
8376	ATCGGTACCGGCACGCTGATCCAGGCGCT	KpnI	pKaS59
8395	ACGGSTCTAGAGGAAGATCTTGGATCACCTCGGTGATGGT	n/a	pKaS77

8396	CAAGATCTTCCTCTAGACCCGTGCAGTCCGCGCAGCAGGT	n/a	pKaS77
8397	TCAAGCTGGCGACCGCGGGATAGCGTCTAGAGCATTTTCC	n/a	pKaS77
8398	GGAAAATGCTCTAGACGCTATCCCGCGGTCGCCAGCTTGA	n/a	pKaS77
8425	GTGGTACCCTCGGCCACACTGCGGACCC	KpnI	pKaS67
8828	GAATTCGAACGTTACGCGTC	EcoRI	pKaS77
8829	GCATGGACGAGCTGTACAAGGGGTCTAGAGGAAGATCT	n/a	pKaS77
8830	AGATCTTCCTCTAGACCCCTTGTACAGCTCGTCCATGC	n/a	pKaS77
8831	AGTGGCTAGCCTATCCCGCGGTCGC	NheI	pKaS77
8890	TGGGTGCGATCGACTTTTGCCACATGATG	n/a	pKaS84
8891	CAAAAGTCGATCGCACCCACGCCGAGCACGT	n/a	pKaS84
9216	AGTGAAGCTTTCACTCGGCCACACTGCGGACCC	HindIII	pKaS93
9432	GTAGGTACCGTGCAGTCCGCGCAGCAGGTCGATGGCGGCCATCGCCTTG	KpnI	pKaS95

References

1. **Jenal U**, **Shapiro L.** 1996. Cell cycle-controlled proteolysis of a flagellar motor protein that is asymmetrically distributed in the *Caulobacter* predivisional cell. EMBO J **15**:2393–406.
2. **Gustafsson MG**. 2000. Surpassing the lateral resolution limit by a factor of two using structured illumination microscopy. J Microsc **198**:82–7.
3. **Schermelleh L**, **Carlton PM**, **Haase S**, **Shao L**, **Winoto L**, **Kner P**, **Burke B**, **Cardoso MC**, **Agard DA**, **Gustafsson MGL**, **Leonhardt H**, **Sedat JW**. 2008. Subdiffraction multicolor imaging of the nuclear periphery with 3D structured illumination microscopy. Science **320**:1332–6.
4. **Cole JL**, **Hardy GG**, **Bodenmiller D**, **Toh E**, **Hinz A**, **Brun YV.** 2003. The HfaB and HfaD adhesion proteins of *Caulobacter crescentus* are localized in the stalk. Mol Microbiol **49**:1671–83.
5. **Schindelin J**, **Arganda-Carreras I**, **Frise E**, **Kaynig V**, **Longair M**, **Pietzsch T**, **Preibisch S**, **Rueden C**, **Saalfeld S**, **Schmid B**, **Tinevez J-Y**, **White DJ**, **Hartenstein V**, **Eliceiri K**, **Tomancak P**, **Cardona A**. 2012. Fiji: an open-source platform for biological-image analysis. Nat Methods **9**:676–682.
6. **Paintdakhi A**, **Parry B**, **Campos M**, **Irnov I**, **Elf J**, **Surovtsev I**, **Jacobs-Wagner C**. 2015. Oufti: An integrated software package for high-accuracy, high-throughput quantitative microscopy analysis. Mol Microbiol 1–11.
7. **Deshpande S**, **Pfohl T**. 2012. Hierarchical self-assembly of actin in micro-confinements using microfluidics. Biomicrofluidics **6**:34120.
8. **Schneider CA**, **Rasband WS**, **Eliceiri KW**. 2012. NIH Image to ImageJ: 25 years of image analysis. Nat Methods **9**:671–5.
9. **Boehm A**, **Steiner S**, **Zaehringer F**, **Casanova A**, **Hamburger F**, **Ritz D**, **Keck W**, **Ackermann M**, **Schirmer T**, **Jenal U**. 2009. Second messenger signalling governs *Escherichia coli* biofilm induction upon ribosomal stress. Mol Microbiol **72**:1500–1516.
10. **Zähringer F**, **Massa C**, **Schirmer T**. 2011. Efficient enzymatic production

of the bacterial second messenger c-di-GMP by the diguanylate cyclase YdeH from *E. coli*. Appl Biochem Biotechnol **163**:71–9.
11. **Christen M**, **Christen B**, **Folcher M**, **Schauerte A**, **Jenal U**. 2005. Identification and characterization of a cyclic di-GMP-specific phosphodiesterase and its allosteric control by GTP. J Biol Chem **280**:30829–37.
12. **Potthoff E**, **Ossola D**, **Zambelli T**, **Vorholt JA**. 2015. Bacterial adhesion force quantification by fluidic force microscopy. Nanoscale **7**:4070–4079.
13. **Grüter RR**, **Vörös J**, **Zambelli T**. 2013. FluidFM as a lithography tool in liquid: spatially controlled deposition of fluorescent nanoparticles. Nanoscale **5**:1097–1104.
14. **Potthoff E**, **Guillaume-Gentil O**, **Ossola D**, **Polesel-Maris J**, **LeibundGut-Landmann S**, **Zambelli T**, **Vorholt JA**. 2012. Rapid and serial quantification of adhesion forces of yeast and Mammalian cells. PLoS One **7**:e52712.
15. **Huang N-P**, **Michel R**, **Voros J**, **Textor M**, **Hofer R**, **Rossi A**, **Elbert DL**, **Hubbell JA**, **Spencer ND**. 2001. Poly(l-lysine)-g-poly(ethylene glycol) Layers on Metal Oxide Surfaces: Surface-Analytical Characterization and Resistance to Serum and Fibrinogen Adsorption. Langmuir **17**:489–498.
16. **Sader JE**, **Chon JWM**, **Mulvaney P**. 1999. Calibration of rectangular atomic force microscope cantilevers. Rev Sci Instrum **70**:3967–3969.
17. **Boehm A**, **Kaiser M**, **Li H**, **Spangler C**, **Kasper CA**, **Ackermann M**, **Kaever V**, **Sourjik V**, **Roth V**, **Jenal U**. 2010. Second messenger-mediated adjustment of bacterial swimming velocity. Cell **141**:107–16.
18. **Spangler C**, **Böhm A**, **Jenal U**, **Seifert R**, **Kaever V**. 2010. A liquid chromatography-coupled tandem mass spectrometry method for quantitation of cyclic di-guanosine monophosphate. J Microbiol Methods **81**:226–231.
19. **Johnson M**, **Zaretskaya I**, **Raytselis Y**, **Merezhuk Y**, **McGinnis S**, **Madden TL**. 2008. NCBI BLAST: a better web interface. Nucleic Acids Res **36**:W5-9.
20. **Alva V**, **Nam S-Z**, **Söding J**, **Lupas AN**. 2016. The MPI bioinformatics Toolkit as an integrative platform for advanced protein sequence and structure analysis. Nucleic Acids Res **44**:W410–W415.
21. **Söding J**, **Biegert A**, **Lupas AN**. 2005. The HHpred interactive server for protein homology detection and structure prediction. Nucleic Acids Res **33**:W244-8.
22. **Hildebrand A**, **Remmert M**, **Biegert A**, **Söding J**. 2009. Fast and accurate automatic structure prediction with HHpred. Proteins Struct Funct Bioinforma **77**:128–132.
23. **Edgar RC**. 2004. MUSCLE: multiple sequence alignment with high accuracy and high throughput. Nucleic Acids Res **32**:1792–7.
24. **Ely B**. 1991. Genetics of *Caulobacter crescentus*, p. 372–384. *In* Methods in Enzymology.
25. **Thanbichler M**, **Iniesta AA**, **Shapiro L**. 2007. A comprehensive set of plasmids for vanillate- and xylose-inducible gene expression in *Caulobacter crescentus*. Nucleic Acids Res **35**:e137.
26. **Khan SR**, **Gaines J**, **Roop RM**, **Farrand SK**. 2008. Broad-Host-Range Expression Vectors with Tightly Regulated Promoters and Their Use To Examine the Influence of TraR and TraM Expression on Ti Plasmid

Quorum Sensing. Appl Environ Microbiol **74**:5053–5062.

27. **Abel S**, **Bucher T**, **Nicollier M**, **Hug I**, **Kaever V**, **Abel Zur Wiesch P**, **Jenal U**. 2013. Bi-modal distribution of the second messenger c-di-GMP controls cell fate and asymmetry during the *Caulobacter* cell cycle. PLoS Genet **9**:e1003744.
28. **Abel S**, **Chien P**, **Wassmann P**, **Schirmer T**, **Kaever V**, **Laub MT**, **Baker TA**, **Jenal U**. 2011. Regulatory cohesion of cell cycle and cell differentiation through interlinked phosphorylation and second messenger networks. Mol Cell **43**:550–60.
29. **Hardy GG**, **Allen RC**, **Toh E**, **Long M**, **Brown PJB**, **Cole-Tobian JL**, **Brun YV**. 2010. A localized multimeric anchor attaches the *Caulobacter* holdfast to the cell pole. Mol Microbiol **76**:409–27.
30. **Toh E**, **Kurtz HD**, **Brun YV**. 2008. Characterization of the *Caulobacter crescentus* holdfast polysaccharide biosynthesis pathway reveals significant redundancy in the initiating glycosyltransferase and polymerase steps. J Bacteriol **190**:7219–31.
31. **Hanahan D**. 1983. Studies on transformation of *Escherichia coli* with plasmids. J Mol Biol **166**:557–80.
32. **Simon R**, **Priefer U**, **Pühler A**. 1983. A broad host range mobilizationsystem for in vivo genetic engineering: transposon mutagenesis in gram negative bacteria. Bio/Technology **1**:784–791.
33. **Evinger M**, **Agabian N**. 1977. Envelope-associated nucleoid from *Caulobacter crescentus* stalked and swarmer cells. J Bacteriol **132**:294–301.
34. **Stove Poindexter JL**, **Cohen-Bazire G**. 1964. The fine structure of stalked bacteria belonging to the family Caulobacteraceae. J Cell Biol **23**:587–607.
35. **Schmidt JM**, **Stanier RY**. 1965. Isolation and Characterization of Bacteriophages Active against Stalked Bacteria. J Gen Microbiol **39**:95–107.
36. **Ely B**, **Johnson RC**. 1977. Generalized transduction in *Caulobacter crescentus*. Genetics **87**:391–399.
